# Machine learning dismantling and early-warning signals of disintegration in complex systems

**DOI:** 10.1038/s41467-021-25485-8

**Published:** 2021-08-31

**Authors:** Marco Grassia, Manlio De Domenico, Giuseppe Mangioni

**Affiliations:** 1 Dip. Ingegneria Elettrica Elettronica e Informatica, Università degli Studi di Catania, Catania, Italy; 2 CoMuNe Lab, Fondazione Bruno Kessler, Povo, TN Italy

**Keywords:** Computer science, Complex networks

## Abstract

From physics to engineering, biology and social science, natural and artificial systems are characterized by interconnected topologies whose features – e.g., heterogeneous connectivity, mesoscale organization, hierarchy – affect their robustness to external perturbations, such as targeted attacks to their units. Identifying the minimal set of units to attack to disintegrate a complex network, i.e. network dismantling, is a computationally challenging (NP-hard) problem which is usually attacked with heuristics. Here, we show that a machine trained to dismantle relatively small systems is able to identify higher-order topological patterns, allowing to disintegrate large-scale social, infrastructural and technological networks more efficiently than human-based heuristics. Remarkably, the machine assesses the probability that next attacks will disintegrate the system, providing a quantitative method to quantify systemic risk and detect early-warning signals of system’s collapse. This demonstrates that machine-assisted analysis can be effectively used for policy and decision-making to better quantify the fragility of complex systems and their response to shocks.

## Abstract (web summary)

Network dismantling allows to find minimum set of units attacking which leads to system’s break down. Grassia et al. propose a deep-learning framework for dismantling of large networks which can be used to quantify the vulnerability of networks and detect early-warning signals of their collapse.

## Introduction

Several empirical systems consist of nonlinearly interacting units, whose structure and dynamics can be suitably represented by complex networks^1^. Heterogeneous connectivity^2^, mesoscale^3,4^, higher-order^5,6^ and hierarchical^7^ organization, efficiency in information exchange^8^, and multiplexity^9–12^ are distinctive features of biological molecules within the cell^13^, connectomes^14^, mutualistic interactions among species^15^, urban^16^, trade^17^, and social^18–20^ systems.

However, the structure of complex networks can dramatically affect its proper functioning, with crucial effects on collective behavior and phenomena such as synchronization in populations of coupled oscillators^21^, the spreading of infectious diseases^22,23^ and cascade failures^24^, the emergence of misinformation^25,26^, and hate^27^ in socio-technical systems or the emergence of social conventions^28^. While heterogeneous connectivity is known to make such complex networks more sensitive to shocks and other perturbations occurring to hubs^29^, a clear understanding of the topological factors—and their interplay—responsible for a system’s vulnerability still remains elusive. For this reason, the identification of the minimum set of units to target for driving a system towards its collapse—a procedure known as network dismantling—attracted increasing attention^30–34^ for practical applications and their implications for policy making. Dismantling is efficient if such a set is small and, simultaneously, the system quickly breaks down into smaller isolated clusters. The problem is, however, NP-hard and while percolation theory provides the tools to understand large-scale transitions as units are randomly disconnected^35–38^, a general theory of network dismantling is missing and applications mostly rely on approximated theories or heuristics.

**Fig. 1:**
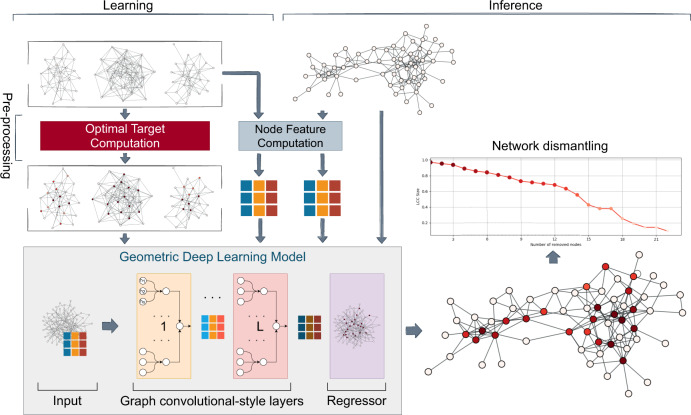
Training a machine to learn complex topological patterns for network dismantling. To build our training data, we generate and dismantle small networks optimally and compute the node features. After the model is trained, it can be fed the target network (again, with its nodes' features) and it will assign each node *n* a value *p*_*n*_, the probability that it belongs to the (sub-)optimal dismantling set. Nodes are then ranked and removed until the dismantling target is reached. The machine learning architecture used consists of graph convolutional-style layers (Graph Attention Network (GAT) layers) coupled with linear layers—that provide residual connections between consecutive layers—followed by a regressor (i.e., a Multilayer Perceptron) with a sigmoid activation function that constrains the *p*_*n*_ value to the [0, 1] range.

Here, we develop a computationally efficient framework—named GDM (Graph Dismantling with Machine learning) and conceptually described in Fig. 1—based on machine learning, to provide a scalable solution, tackle the dismantling challenge, and gain new insights about the latent features of the topological organization of complex networks. Specifically, we employ graph convolutional-style layers, overcoming the limitations of classic (Euclidean) deep learning and operate on graph-structured data. These layers, inspired by the convolutional layers that empower most of the deep-learning models nowadays, aggregate the features of each node with the ones found in its neighborhood by means of a learned nontrivial function, producing high-level node features. While the machine is trained on identifying the critical point from dismantling of relatively small systems—that can be easily and optimally dismantled—we show that it exhibits remarkable inductive capabilities, being able to generalize to previously unseen nodes and way larger networks after the learning phase.

This work follows and combines two recent trends in Machine Learning: learning on synthetic data and generalizing to real-world instances^39^, and learning heuristics to tackle/solve hard combinatorial problems on graphs^40,41^. While the motivation behind the latter is easy to understand, as—thanks to the increasing availability of data—graphs are becoming larger and larger and many interesting applications would be unfeasible due to computational constraints, the idea of learning on synthetic data can be motivated by the unlimited availability of (easily) generated examples with training labels. Thanks to their inductive capabilities and extensive training, deep-learning models trained on synthetic data are able to generalize to real-world instances, providing a useful tool to approach hard problems in general.

## Results

The machine learning framework proposed here consists of a (geometric) deep-learning model, composed of graph convolutional-style layers and a regressor (a multilayer perceptron), that is trained to predict attack strategies on small synthetic networks—that can be easily and optimally dismantled—and then used to dismantle large networks, for which the optimal solution cannot be found in reasonable time. To give an insight, the graph convolutional-style layers aggregate the features of each node with the ones found in its neighborhood by means of a learned nontrivial function, as they are inspired by the convolutional layers that empower most of the (Euclidean) deep-learning models nowadays. More practically, the (higher-order) node features are propagated by the neural network when many layers are stacked: deeper the architecture, i.e., the more convolutional layers, the farther the features propagate, capturing the importance of the neighborhood of each node. Specifically, we stack a variable number of state-of-the-art layers, namely Graph Attention Networks (GAT)^42^, that are based on the self-attention mechanism (also known as intra-attention), which was shown to improve the performance in natural language processing tasks^43^. These layers are able to handle the whole neighborhood of nodes without any sampling, which is one of the major limitations of other popular convolutional-style layers (e.g., GraphSage^44^), and also to assign a relative importance factor to the features of each neighboring node that depends on the node itself thanks to the attention mechanism.

Such detailed model takes as input one network at a time plus the features of its nodes and returns a scalar value *p*_*n*_ between zero and one for every node *n*. During the dismantling of a network, nodes are sorted and removed (if they belong to the LCC) in descending order of *p*_*n*_ until the target is reached.

### Dismantling synthetic and real-world systems

In our experiments, we dismantle empirical complex systems of high societal or strategic relevance (e.g., biological, social, infrastructure, communication, trophic, and technological systems), our main goal being to learn an efficient attack strategy. To validate the goodness of such a strategy, we compare against state-of-the-art dismantling methods, such as Generalized Network Dismantling (GND)^34^, Explosive Immunization (EI)^45^, CoreHD^46^, Min-Sum (MS)^33^, and Collective Influence (CI)^32^, using local (node degree and its *χ*^2^ value over the neighborhood), second-order (local clustering coefficient), and global (*k*–core value) node features as input features.

To quantify the goodness of each method in dismantling the network, we consider the Area Under the Curve (AUC) encoding changes in the Largest Connected Component (LCC) size across the attacks. The LCC size is commonly used in the literature to quantify the robustness of a network, because systems need the existence of a giant cluster to work properly. The AUC indicator has the advantage of accounting for how quickly, overall, the LCC is disintegrated: the lower the area under the curve, the more efficient is the network dismantling. We compute the AUC value by integrating the *LCC*(*x*)/∣*N*∣ values using Simpson’s rule.

**Fig. 2:**
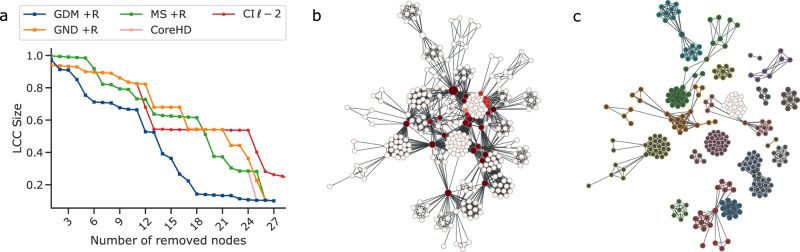
Dismantling the Brazilian corruption network. **a** GDM and state-of-the-art algorithms with reinsertion of the nodes are compared. The network before (**b**) and after (**c**) a GDM attack is shown. The color of the nodes represents (from dark red to white) the attack order, while their size represents their betweenness value. In the attacked network, darker nodes do not belong to the LCC, and their contour color represents the component they belong to.

As a representative example, we show in Fig. 2a the result of the dismantling process for the corruption network^47^, built from 65 corruption scandals in Brazil, as a function of the number of removed units. Results are shown for GDM and the cutting-edge algorithms mentioned above. In Fig. 2b, c, instead, we show the structure before and after dismantling, respectively. Our framework disintegrates the network faster than other methods: to verify if this feature is general, we perform a thorough analysis of several empirical systems.

**Fig. 3:**
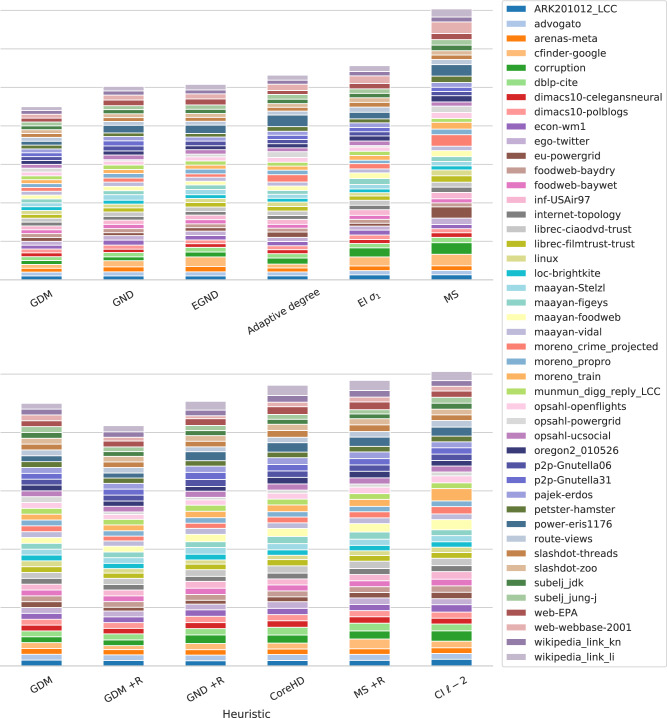
Dismantling empirical complex systems. Per-method cumulative area under the curve (AUC) of real-world networks dismantling. The lower the better. The dismantling target for each method is 10% of the network size. Each value is scaled to the one of our approach (GDM) for the same network. GND stands for Generalized Network Dismantling, EGND for Ensemble approach for GND (in both GND and EGND, cost matrix **W** = **I**), MS stands for Min-Sum, EI *σ*_1_ stands for Explosive Immunization (*σ*_1_) algorithm and CI for Collective Influence. +R means that the reinsertion phase is performed. CoreHD and CI are compared to other +R algorithms as they include the reinsertion phase. Also, note that some values are clipped (limited) to 3× for the MS heuristic to improve visualization.

Figure 3 shows the performance of each dismantling method on each empirical system considered in this study, allowing for an overall comparison. On average, our approach outperforms the others. For instance, Generalized Network Dismantling’s cumulative AUC is ~12% higher and the Min-Sum algorithm is outscored by a significant margin, which is remarkable considering that our approach is static—i.e., predictions are made at the beginning of the attack—while the other ones are dynamic—i.e., structural importance of the nodes is (re)computed during the attacks. For a more extensive comparison with these approaches, we also introduce a node reinsertion phase using a greedy algorithm which reinserts, a posteriori, those nodes that belong to smaller components of the (virtually) dismantled system and which removal is not actually needed in order to reach the desired target^33^. Once again, our approach outperforms the other algorithms: even without accounting for the reinsertion phase, GDM performs comparably with GND + reinsertion and outscores the others, highlighting how it is able to identify the more critical nodes of a network.

**Fig. 4:**
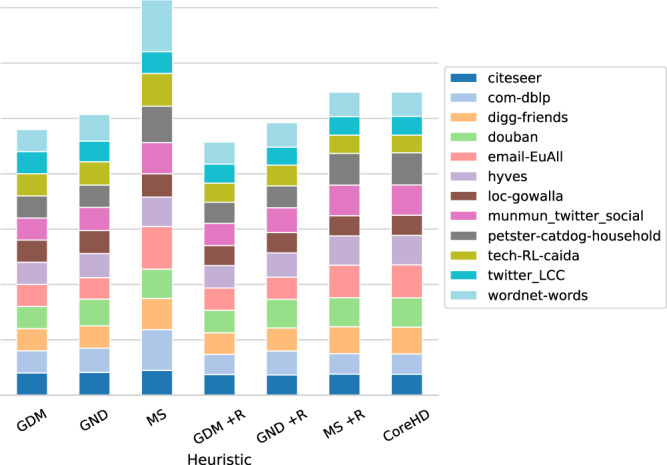
Dismantling empirical complex large systems. Per-method cumulative area under the curve (AUC) of real-world networks dismantling. The lower the better. The dismantling target for each method is 10% of the network size. We compute the AUC value by integrating the *LCC*(*x*)/∣*N*∣ values using Simpson’s rule, and each value is scaled to the one of our approach (GDM) for the same network. GND stands for Generalized Network Dismantling (with cost matrix **W** = **I**) and MS stands for Min-Sum. +R means that the reinsertion phase is performed. Also, note that some values are clipped (limited) to 3× for the MS heuristic to improve visualization.

We extend the comparison against the more promising state-of-the-art algorithms (GND and MS with and without reinsertion, and CoreHD) to 12 large networks with up to 1.8M nodes and up to 2.8M edges. As shown in Fig. 4, the results on smaller empirical networks are confirmed even for the large ones, although with smaller margins (i.e., ~5.6% and ~7.6% against GND, respectively, with and without the reinsertion phases). This is still impressive as the proposed approach is static while the others recompute the nodes’ structural importance during the dismantling process, which involves many removals for these networks (e.g., 70K on hyves network) and changes the network topology drastically, confirming the validity of our approach.

**Fig. 5:**
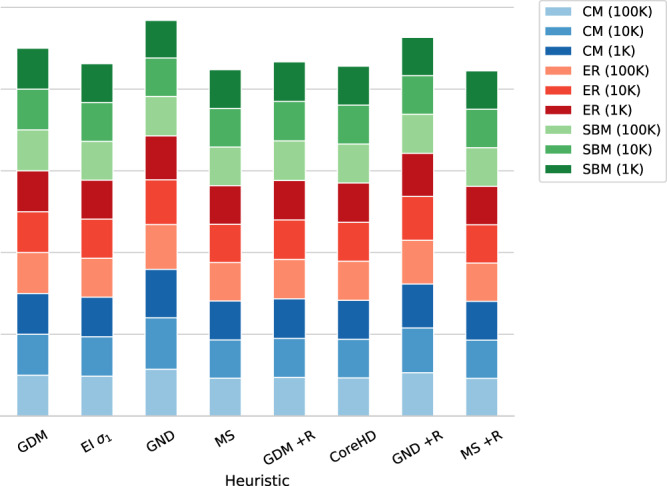
Dismantling synthetic complex systems. Per-method cumulative area under the curve (AUC) of the dismantling of synthetic networks. The lower the better. Each value is the average on 10 different instances, and is scaled to the AUC of our approach (GDM) for the same network type. CM stands for Configuration Model, ER stands for Erdős-Rényi, and SBM stands for Stochastic Block Model.

We also test synthetic networks—i.e., Erdős-Rényi (ER), on Configuration Model networks (CM) with power law distribution and Stochastic Block Model (SBM). As reported in Fig. 5, the best approach is Min-Sum, scoring 6% and 3% lower AUC than GDM and GDM + R, respectively. The reason behind this slightly lower GDM performance can be found in our training set and on what the models learn. Specifically, we train on networks generated using three different models, which teaches the models to look for patterns that turn out to be suboptimal in the long term (as no recomputation is made during the process) when it comes to specific synthetic networks. It should also be noted that GND—the second best-performing algorithm on real-world networks—is the worst of the tested algorithms on synthetic networks.

We refer the reader to Supplementary Figs. 5 and 6 for the full dismantling curves (i.e., LCC as a function of the removed nodes), to the Supplementary Tables 1 and 3 for the numerical results of all the experiments, and to Supplementary Table 2 for the extensive list of the real-world test networks.

An interesting feature of our framework is that it can enhance existing heuristics based on node descriptors, by employing the same measure as the only node feature, as shown in Supplementary Fig. 3.

We stress that the node features used in this work are arbitrary. In fact, while we selected them to keep low the computational complexity of the dismantling process, the graph convolutional networks (and, therefore, GDM) can process any node feature combination. That is, if better dismantling performance are required, more complex ones can be chosen.

### Understanding the models

After validating the dismantling performance of our approach, an investigation of what the models are actually learning and how they are making the long-term predictions is needed to open the black box of deep learning and use the resulting insights to improve the state-of-the-art algorithms.

For this purpose, we employ GNNExplainer^48^, the novel framework for explaining graph convolutional-style networks, to extract the explanation subgraphs (the subsets of nodes and edges) that most account for the value predicted by the model for each node. What we find in the analysis of the explanation subgraphs of the networks in our test-set is that, as shown for the Brazilian corruption network in the Supplementary Information, the model is removing the nodes that bridge multiple clusters, discovered by combining the input features and by looking to other bridges in their *K*-hop neighborhood, which confirms the insight provided by the toy-examples discussed in the Supplementary Information. The identification of this kind of bridges is achieved thanks to the local and second-order features combined with the propagation performed by the model. In fact, while Lauri et al.^41^ show that the degree, its *χ*^2^ value over the neighborhood and the local clustering coefficient can be used to estimate the likelihood a node belongs to a clique via classical deep-learning tools, our geometric deep-learning model improves the idea by extending the feature propagation in a *K*-hop radius and the result is improved further by the *k*–core value that helps to filter the nodes at the core of the network. Although some of the targeted nodes are not the direct cause of large damage to the network, they are needed to drive the network in a vulnerable state where the removal of other nodes disrupts it. In other words, the models seem to predict a long-term strategy that aims not only to remove the Articulation Points (AP, also known as Cut Vertices, are nodes that, when removed, cause the creation of new connected components) but also create new ones with the removal of other non-AP nodes.

**Fig. 6:**
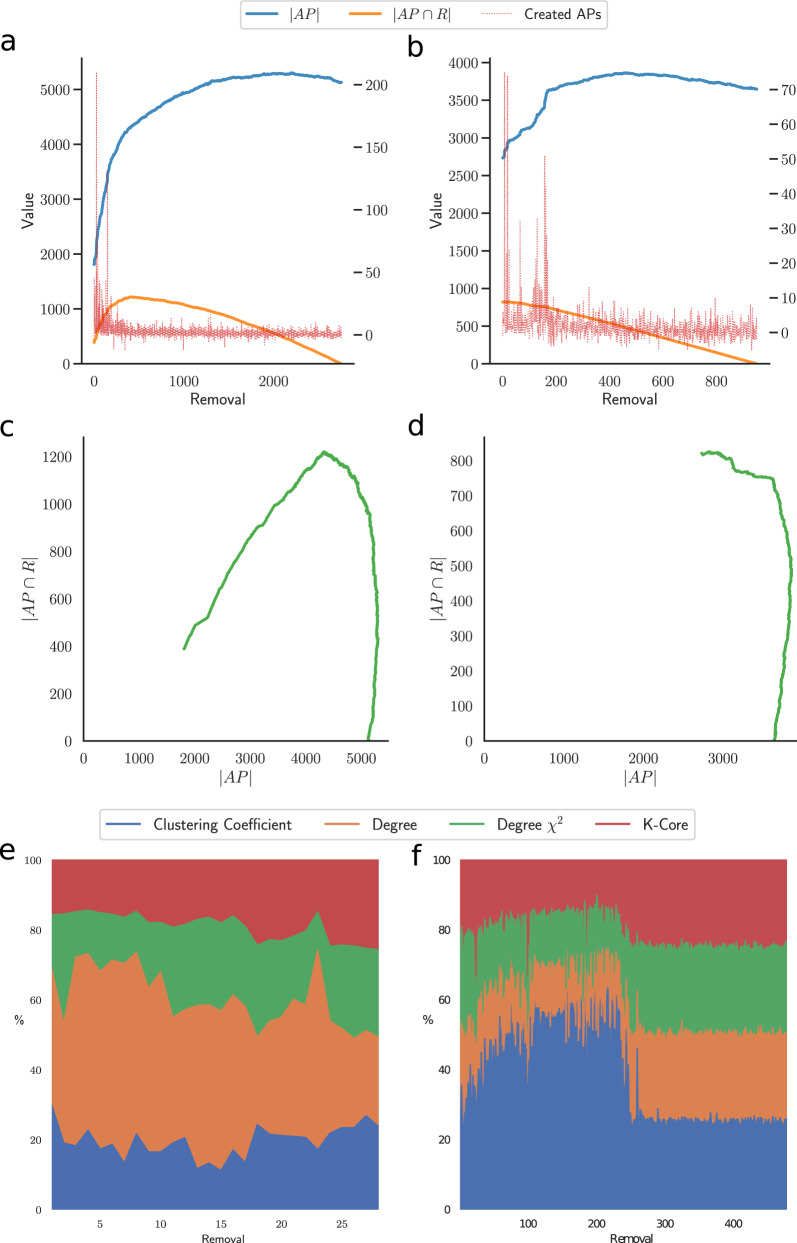
Understanding our models. The analysis of the Articulation Points of the networks (*AP*) and how many of them are in the removal list (*R*) shows that the models are learning a long-term strategy that aims to create new articulation points and remove the ones that deal most damage to the network. As an example, we show the linux (**a**, **c**) and the internet-topology (**b**, **d**) networks. This is achieved using the input node features discussed above, that allow the identification of clusters and bridges. We show an example of the relative importance of the node features for the corruption (**e**) and for the subelj-jdk (**f**) networks.

This insight led us to investigate further in this direction with an analysis of the Articulation Points as the nodes are removed. Specifically, we compute, removal after removal, the number of APs in the network and how many of them are in the removal list (*R*) predicted by the model. As shown in Fig. 6a, b for the linux and internet-topology networks, the number of APs increases as nodes are removed, and so do the ones in the removal list, until there is a natural decay due to the decreasing size of the removal list itself. This trend is confirmed for most of our test networks, as shown in Supplementary Fig. 9.

Considering the high dismantling performance, this proves that not only the model is effectively learning to target the nodes that cause the network collapse when removed together, but also that does so more efficiently than other algorithms. Note that a strategy barely based on AP removal would not be effective, since an AP can be one node whose removal separates a giant connected component from a component consisting of a negligible number of nodes (e.g., only one node). Instead, we demonstrate that our model is learning to identify the most effective AP for disintegrating the target system: elegantly, these ones turn out to be bridges between large clusters, not between one large and one small cluster.

Moreover, if we analyze the number of APs in the removal list (∣*AP* ∩ *R*∣) as a function of the total number of APs (∣*AP*∣), we find that the two are related by a kind of deterministic dynamics, resembling the one which characterizes chaotic systems and, specifically, chaotic maps such as the logistic map or the Hénon map, where parabolic attractors emerge when the state of the system at the *n* + 1th step is plotted against the state at the *n*th step. In our case, the *n*th step coincides with the removal of the *n*th node in the removal list. The shape of the resulting attractor provides a strong characterization of the system and its robustness: we show an example for each type in Fig. 6c, d (more examples can be found in Supplementary Fig. 10). That is, in the first case, the model drives the network in a state where the nodes in the removal list become Articulation Points, in the latter it mainly removes nodes that are already APs.

After understanding what the model is learning, we analyze how features account in the computation of the output values to get an insight on how the model selects the nodes. While there is no prevailing feature for all the networks—e.g., sometimes the degree is the key feature, others the *K*–core value, etc.—an interesting result is that the feature weight also changes with the score of the nodes. For instance, while the clustering coefficient is the main feature, scoring up to the 60% of the relative importance, in the first 250 removals of the subelj-jdk network (Fig. 6f), all the features gain equal weight after that removal. In the Brazilian corruption network, instead, the node degree is the most important feature to identify the first nodes to remove, but other features gain more importance to identify less important nodes, needed to reach the dismantling target. These results confirm that the definition of new algorithms based on these insights is extremely hard, as the weight of each feature is adapted by the model to the topology and to the patterns in the network. At this point, it is plausible to assess that our framework learns correlations among node features. To probe this hypothesis, in Supplementary Fig. 4 we analyze the configuration models of the same networks analyzed so far: those models keep the observed connectivity distribution while destroying topological correlations. We observe that the dismantling performance drops on these models, confirming that the existing topological correlations are learned and, consequently, exploited by the machine.

For more insights, details about the implementation and the information about the tools used, we refer the reader to the Supplementary Information.

### Early-warning signals of systemic collapse

Another relevant output of our method is the calculation of a damage score that can be used to predict the impact of future attacks to the system. Accordingly, we introduce an estimator of early warning that can be used for inform policy and decision-making in applications where complex interconnected systems—such as water management systems, power grids, communication systems and public transportation networks—are subject to potential failures or targeted attacks. We define Ω, namely early warning, as a value between 0 and 1, calculated as follows. We first simulate the dismantling of the target network using our approach and call *S*_*o*_ the set of virtually removed nodes that cause the percolation of the network. Then, we sum the *p*_*n*_ values predicted by our model for each node *n* ∈ *S*_*o*_ and define

1 $${{{\Omega }}}_{m}=\mathop{\sum}\limits_{n\in {S}_{o}}\ {p}_{n}$$

The value of the early-warning Ω for the network after the removal of a generic set *S* of nodes is given by

2 $${{\Omega }}=\left\{\begin{array}{ll}{{{\Omega }}}_{s}/{{{\Omega }}}_{m}&\,{{\mbox{if}}}\,\,{{{\Omega }}}_{s}\,\le\, {{{\Omega }}}_{m}\\ 1&\,{{\mbox{otherwise}}}\,\end{array}\right.$$

where $${{{\Omega }}}_{s}=\mathop{\sum}\nolimits_{n\in S}{p}_{n}$$.

The rationale behind this definition is that the system will tolerate a certain amount of damage before it collapses: this value is captured by Ω_*m*_. Ω will quickly reach values close to 1 when nodes with key-role in the integrity of the system are removed. Of course, the system could be heavily harmed by removing many less relevant nodes (e.g., the peripheral ones) with an attack that causes a small decrease in LCC size over time, and probably get a low value of Ω. However, this kind of attacks does not need an early-warning signal since they do not cause an abrupt disruption of the system and can be easily detected.

**Fig. 7:**
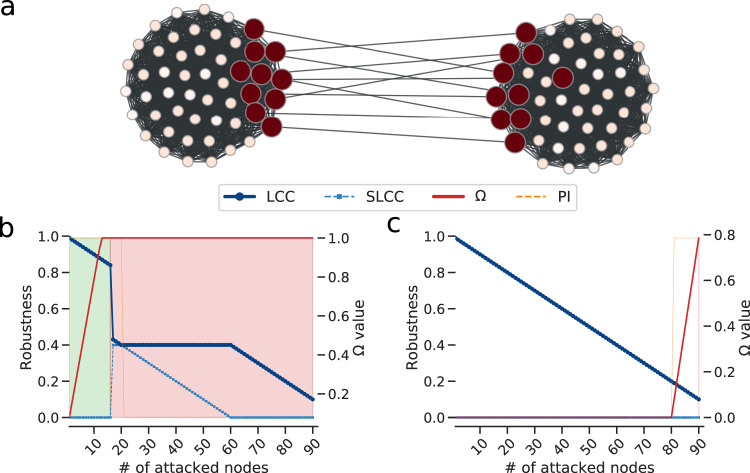
Why do we need an early-warning signal? Toy-example meant to explain why the LCC is not sufficient to evaluate the state of the system: in (**a**) we show a toy-example network composed of two cliques connected by 10 bridges. The size of the nodes represents their betweenness value and the color (from dark red to white) represents their importance to the system’s health according to our method. As illustrated in (**b**) and (**c**), the LCC decreases at the same rate during the initial part of both a betweenness and an inverse betweenness-based attacks. Instead, Ω values do not and reach warning levels before the system suddenly collapses. Note that LCC and SLCC are the largest and second-largest connected components respectively, Ω is the early-warning descriptor introduced in this study, and PI is the *p*_*n*_ value of each removed node.

Why do we need an early-warning signal? In Fig. 7 we show a toy-example meant to explain why the Largest Connected Component size may not be enough to determine the state of a system. The toy-example network in Fig. 7a is composed of two cliques (fully connected subnetworks) connected by a few border nodes (bridges) that also belong to the respective cliques. Many dismantling approaches (like the degree and betweenness-based heuristics, or even ours) would remove those bridge nodes first, meaning that the network would eventually break in two, as shown in Fig. 7b. Now, when most of the bridge nodes are removed (e.g., after 16 removals), the LCC is still quite large as it includes more than 80% of the nodes, but it takes just a few more removals of the bridges to break the network in two. While Ω is able to capture the imminent system disruption (i.e., the Ω value gets closer to 1 very fast), the LCC size is not, and one would notice when it is too late. Moreover, the LCC curve during the initial part of the attack is exactly the same as the one in Fig. 7c, showing the removal of nodes in inverse degree (or betweenness) order, which does not cause the percolation of the system. Again, Ω captures this difference and does not grow, meaning that a slow degradation should be expected.

**Fig. 8:**
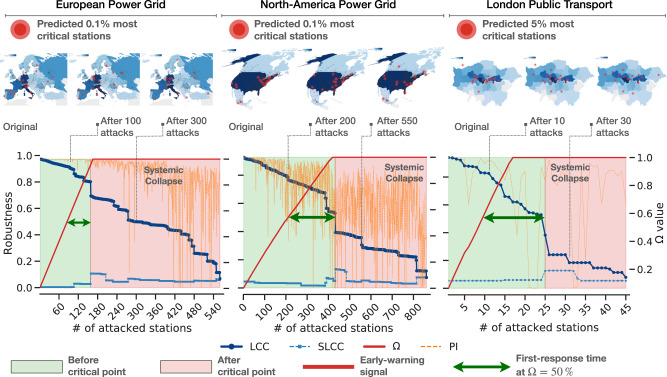
Early warning due to network dismantling of real infrastructures. Three empirical systems, namely the European power grid (left), the North-American power grid (middle) and the London public transport (right), are repeatedly attacked using a degree-based heuristics, i.e., hubs are damaged first. A fraction of the most vulnerable stations is shown for the original systems and some representative damaged states (i.e., before and after the critical point for system’s collapse), in the top of the figure. The plots show the behavior of the largest (LCC) and second-largest (SLCC) connected components, as well as the behavior of Ω, the early-warning descriptor introduced in this study and the *p*_*n*_ value of each removed node (PI). Transitions between green and red areas indicate the percolation point of the corresponding systems, found through the SLCC peak. We also show the first response time in arbitrary units (arb. units), to highlight how our framework allows to anticipate system’s collapse, allowing for timely emergency response.

We test our method on key infrastructure networks and predict the collapse of the system under various attack strategies (see Fig. 8 for details). Remarkably, while the LCC size decreases slowly without providing a clear alarm signal until the system is heavily damaged and collapses, Ω grows faster when critical nodes are successfully attacked, reaching warning levels way before the system is disrupted, as highlighted by the First Response Time, defined as the time occurring between system’s collapse and an early-warning signal of 50% (i.e., Ω = 0.5). Moreover, the first order derivative ${{\Omega }}^{\prime}$ tracks the importance of nodes that are being attacked, providing a measure of the attack intensity over time.

## Discussion

Our results show that using machine learning to learn network dismantling comes with a series of advantages. While the ultimate theoretical framework is still missing, our framework allows one to learn directly from the data, at variance with traditional approaches, which rely on the definition of new heuristics, metrics or algorithms. An important advantage of our method, typical of data-driven modeling, is that it can be further improved by simply retuning the parameters of the underlying model and training again: conversely, existing approaches require the (re)definition of heuristics and algorithms which are more demanding in terms of human efforts. Remarkably, the computational complexity of dismantling networks with our framework is considerably low: just *O*(*N* + *E*), where *N* is system’s size and *E* the number of connections—which drops to *O*(*N*) for sparse networks (for more information about the computational complexity, see the dedicated section of the Supplementary information). This feature allows for applications to systems consisting of millions of nodes while keeping excellent performance in terms of computing time and accuracy. We also provide deep-insights about the models that should help to understand the power of geometric deep learning. Last but not least, from a methodological perspective, it is worth remarking that our framework is general enough to be adapted and applied to other interesting NP-hard problems on networks, opening the door for new opportunities and promising research directions in complexity science, together with very recent results employing machine learning, for instance, to predict extreme events^49^.

The impact of our results is broad. On the one hand, we provide a framework which disintegrates real systems more efficiently and faster than state-of-the-art approaches: for instance, applications to covert networks might allow hindering communications and information exchange between harmful individuals. On the other hand, we provide a quantitative descriptor of damage which is more predictive than existing ones, such as the size of the largest connected component: our measure allows to estimate the potential system’s collapse due to subsequent damages, providing policy and decision makers with a quantitative early-warning signal for triggering a timely response to systemic emergencies, for instance in water management systems, power grids, communication, and public transportation networks.

## Methods

### Training methodology

We train our models in a supervised manner. Our training data are composed of small synthetic networks (25 nodes each) generated using the Barabási-Albert (BA), the Erdős-Rényi (ER), and the Static Power law generational models that are implemented in igraph^50^ and NetworkX^51^. Each synthetic network is dismantled optimally using brute-force and nodes are assigned a numeric label (the learning target) that depends on their presence in the optimal dismantling set(s). That is, we find all the minimum size solutions using brute-force (i.e., we try all the combinations of nodes) that reduce the Largest Connected Component (LCC) to a given target size, ~18% in our tests; then, the label of each node is computed as the number of optimal sets it belongs to, divided by the total number of optimal solutions. For example, if there is only a set of optimal size, we assign a label value of 1 to the nodes in that set and 0 to all other nodes; if there are two optimal solutions, we assign 1 to the nodes that belong to both sets, 0.5 to the ones that belong to a single set and 0 to all the others. This is meant to teach the model that some nodes are more critical than others since they belong to many optimal dismantling sets.

We stress that the training label is arbitrary and others may work better for other training sets or targets. Moreover, while we train on a generic purpose dataset that includes both power law and ER networks, the training networks can also be chosen to fit the target networks, e.g., by using networks from similar domains or with similar characteristics.

### Model parameters

We run a grid search to test various combination of model parameters, which are reported here, and select the models that better fit the dismantling target (i.e., lower area under the curve or lower number of removals).

- Convolutional-style layers: Graph Attention Network layers.
  - Number of layers: from 1 to 4;
  - Output channels for each layer: 5, 10, 20, 30, 40, or 50, sometimes with a decreasing value between consecutive layers;
  - Multi-head attentions: 1, 5, 10, 15, 20, or 30 concatenated heads;
  - Dropout probability: fixed to 0.3;
  - Leaky ReLU angle of the negative slope: fixed to 0.2;
  - Each layer learns an additive bias;
  - Each layer is coupled with a linear layer with the same number of input and output channels;
  - Activation function: Exponential Linear Unit (ELU). The input at each convolutional layer is the sum between the output of the GAT and the linear layers;
- Regressor: multilayer perceptron
  - Number of layers: from 1 to 4;
  - Number of neurons per layer: 20, 30, 40, 50, or 100, sometimes with a decreasing value between consecutive layers.
- Learning rate: fixed to 10^−5^;
- Epochs: we train each model for 50 epochs;

## Supplementary information

Supplementary Information

## Data Availability

The synthetic data generated in this study has been deposited in the Zenodo database available at 10.5281/zenodo.5105912^52^.

## Code availability

The code of the GDM framework proposed in this paper is available on GitHub at https://github.com/NetworkScienceLab/GDM and on Zenodo at 10.5281/zenodo.5105912^52^.
