# Supplementary material for: Machine learning dismantling and early-warning signals of disintegration in complex systems
Source: Nat Commun. 2021 Aug 31;12:5190. doi: 10.1038/s41467-021-25485-8 (PMC8408155; doi:10.1038/s41467-021-25485-8)
Supplement: Supplementary file 1 — Supplementary Information [file 41467_2021_25485_MOESM1_ESM.pdf]

Supplementary Information for Machine learning dismantling and early-warning signals of disintegration in complex systems

Marco Grassia[1], Manlio De Domenico[2*], Giuseppe Mangioni[1*]

[1]Dip. Ingegneria Elettrica, Elettronica e Informatica - Università degli Studi di Catania - Italy
[2]CoMuNe Lab, Fondazione Bruno Kessler, Via Sommarive 18, 38123 Povo (TN), Italy

*To whom correspondence should be addressed;
E-mail: giuseppe.mangioni@dieei.unict.it; mdedomenico@fbk.eu.

Supplementary Note 1: Deep Learning Model

How the model works

A simplistic but more practical understanding of how a model with L GAT network layers assigns the p_n value to each node n can be achieved by considering the L-hop neighborhood of the node. Consider a tree with height L with node n as root and where each node's neighbors are its children. That is, each level $l + 1$ of the tree is populated with the neighbors of nodes at level l. For instance, at level 1 we have n's neighbors.

Now, n's high-level node features (h_n^L) are computed by aggregating the information from the L-hop neighborhood in a bottom up fashion. Each GAT network layer processes a level of the three, so the deeper the model, the farther the information comes from. That is, the model starts from the bottom of the tree (i.e., the nodes at L hops from n) to compute the high-level node features of each node at layer L and goes up until the root (node n) is reached.

This means that the model is able to aggregate the information in the whole n's L-hop neighborhood in h_n^L, which also accounts for the different importance each node has in that neighborhood thanks to the GAT's self-attention mechanism. The basic idea is somehow similar to the Collective Influence approach, with the main differences being that the geometric deep learning model learns a weighted sum function from the training data to aggregate many node features, whereas the Collective Influence just sums the degrees, and also that the model aggregates the whole L-hop neighborhood ball, not just its frontier.

These high-level features (h_n^L) are then fed to a regressor that returns p_n, the node's structural importance indicator used in our work.

The actual implementation of our model relies on PyTorch Geometric library (*1*) on-top of PyTorch (*2*), while the handling of the graphs (i.e., implementation of the data structures, removal of the nodes and the computation of the connected components) is performed using graph-tool (*3*).

Node features

Considering that the model can process any feature combination, one could just choose to stuff every suitable node metrics that comes to his mind and, since it is proven that Deep Neural Networks learn the feature importance, let them do the rest. On the other hand, it could also be tempting to use no features at all (e.g, a constant value for every node) since Kipf et al. (*4*) showed that their Graph Convolutional Network (GCN), a particular type of convolutional-style graph neural networks, can learn to linearly separate the communities based on the network structure alone and on minimal supervision (one labelled node per community), meaning that convolutional-style neural networks can leverage the network topology to assign a higher-level node feature that describes its role in the network.

We argue that, while the first idea could make sense for scenarios where training data is

abundant and the features are cheap to compute, and while the second shows worse (with respect to models with simple features) but still interesting performance, it makes sense to perform some feature selection a priori to keep the computational complexity of the attack low and also to speed-up the learning process. With that in mind, we pick node degree (plus its chi-square value over the neighborhood[1] over the local neighborhood), k–coreness and local clustering coefficient as node features.

Computational complexity

The computational complexity of our approach mainly depends on two elements: 1) the computational complexity of the node features used and 2) the computational complexity of the convolutional-style layers in the model. In particular, the convolutional-style layers that we employ, i.e., the Graph Attention Networks, scale as $O(N + E)$ where N is the number of nodes and E is the number of edges in the network. Considering that real-world networks are usually sparse, we assume that $O(E) \approx O(N)$, so $O(N + E) \approx O(N)$, and the computational complexity of our approach is the maximum between this and the computational complexity of the features. Given that, the most expensive feature we compute in our experiments is the k–coreness, that is $O(N + E)$, so the computational complexity of the approach detailed above is $O(N)$. For what concerns the computational complexity of the brute-force performed during the training set generation, it is irrelevant as it is a highly parallelizable one-time task that is performed on very small networks. Moreover, since the neural models can generalize, there is no need to train them for each dismantling, and the actual time spent training is negligible.

[1]The chi-square value of the degree of node i is computed as $\chi_i^2 = (\mathbb{E}[d] - \sigma_d)^2 / \mathbb{E}[d]$, where d is the degree of neighboring nodes.

Supplementary Note 2: Understanding GDM's behavior

Before testing on real-world networks, we investigate the behavior of our approach by dismantling some toy-example networks. To this aim, we employ the same low computational complexity node features from the main paper (that are also detailed above).

The first toy example, shown in Supplementary Figure 1a, is a network built from three ego-networks joined by a bridge. The betweenness based heuristics[2], and also our common sense, would suggest removing the bridge first, reducing the LCC size to one third of the initial value, and then remove the nodes at the center of the unconnected ego networks left, for a total of four removals. Instead, our model predicts a different strategy and removes only the cores of the ego sub-networks, reaching the same LCC size with just three removals, as shown in Supplementary Figure 2a.

At this point, we want to probe if the model is just learning to remove the nodes in descending degree order as the previous example would suggest. If that is the case, in our second toy example network, composed of a clique with an appended tail as illustrated in Supplementary Figure 1b, the model would remove the nodes in the clique first, given their high degree. Instead, the tail is detached first, meaning that the predicted strategy differs from the degree based one, and both the degree and betweenness-based heuristics are outperformed, as shown in Supplementary Figure 2b.

Supplementary Note 3: Synthetic networks

As already discussed in the main paper, we also test our approach on synthetic networks. Here, we detail the parameters used to generate the networks. Specifically, we test on Erdős-Rényi (ER) networks (average degree $k_{avg} = 4$), on Configuration Model networks (CM) with power

[2] The removal of nodes by descending betweenness centrality order. The node betweenness is a node centrality measure that captures the importance of the node to the shortest paths through the network.

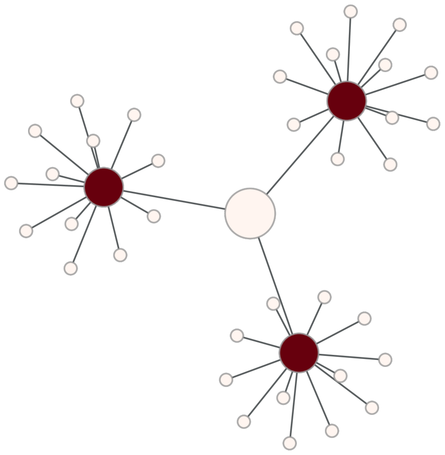

(a) Three bridged ego networks.

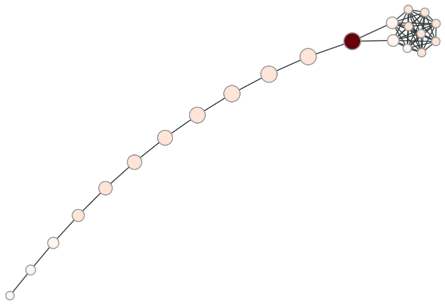

(b) Tailed clique

Supplementary Figure 1: Toy examples. The color of the nodes represents (from dark red to white) the removal order of predicted strategy, while their size represents their betweenness value.

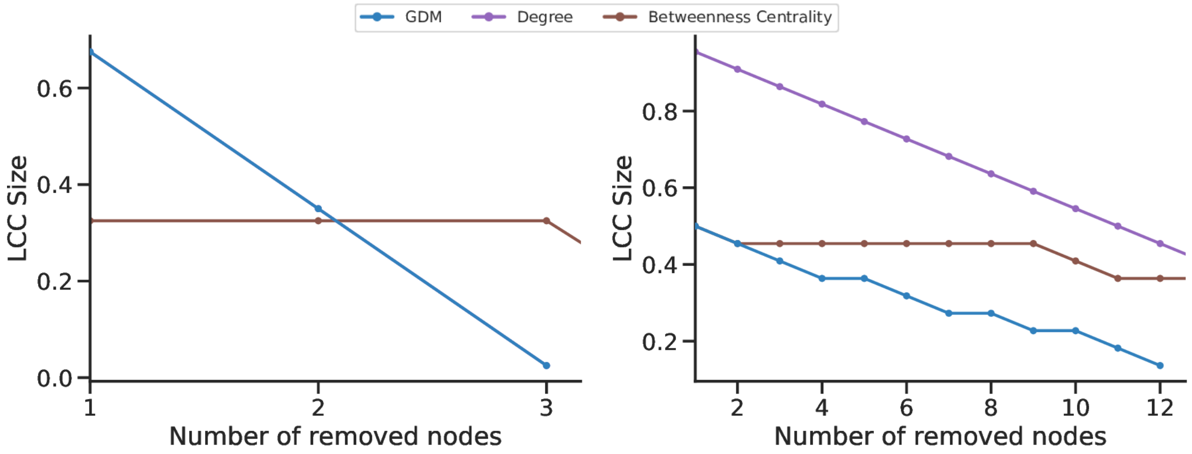

(a) Three bridged ego networks.

(b) Tailed clique

Supplementary Figure 2: Dismantling the toy example networks using our approach, GDM, and the degree and betweenness based heuristics as comparison.

law distribution ($\gamma = 2.5$ and $k_{avg} = 4$) and on Stochastic Block Model (SBM) networks (group size fixed to 100, $p_{intra} = 0.1$ and $p_{inter} = \frac{5}{|N|}$). We generate 10 realizations with $1K$, $10K$ and $100K$ nodes each and average the results. We report the results in numerical form in Supplementary Table 1.

Heuristic<br>Network	GDM	EI σ_1	GND	MS	GDM +R	CoreHD	GND +R	MS +R
CM (100K)	100.0	97.3	114.5	92.6	94.5	93.2	106.0	92.3
CM (10K)	100.0	96.7	126.1	93.9	95.5	94.5	109.5	93.6
CM (1K)	100.0	96.9	118.2	95.1	96.6	95.6	107.5	94.8
ER (100K)	100.0	95.5	109.7	93.9	96.6	95.4	107.2	93.8
ER (10K)	100.0	95.3	109.5	93.9	96.6	95.6	106.9	93.7
ER (1K)	100.0	95.5	107.3	94.5	96.6	95.8	105.4	94.2
SBM (100K)	100.0	95.0	96.9	94.5	96.7	95.2	96.0	94.1
SBM (10K)	100.0	94.8	94.0	94.5	96.7	95.3	94.4	94.1
SBM (1K)	100.0	95.2	91.9	94.9	96.9	95.4	93.5	94.4
Average	100.0	95.8	107.6	94.2	96.3	95.1	102.9	93.9

Supplementary Table 1: Synthetic network results table. Per method area under the curve (AUC) of the dismantling of synthetic networks. The lower the better. Each value is the average on 10 different instances, which is scaled to the AUC of our approach (GDM) for the same network type.

Supplementary Note 4: Dismantling real-world complex systems

Enhancement of metric-based heuristics

In order to better understand how our framework is able to outperform cutting-edge algorithms, we compare existing node metric-based heuristics (e.g., removal of nodes in degree order) against GDM models that employ the corresponding node metric as the only node feature. As an example, in Supplementary Figure 3 we display the enhancement of the degree and the betweenness based heuristics in the left and right columns respectively. These GDM-enhanced heuristics effectively outperform the vanilla ones, highlighting the fact that the model is able to capture the importance of the nodes thanks to the feature propagation discussed before. This also gives an important insight as the model seems to learn correlations between node features.

Dismantling of configuration model rewired networks

We investigate further if the model is learning correlations among node features by dismantling the configuration model rewirings[3] of the networks in our test set. If that is the case, the dismantling power of our approach on the rewirings should be heavily affected. In Supplementary Figure 4 we show, for each network, the dismantling of 1000 configuration models and also the original instance as comparison. In all the tested networks, there is a severe performance drop. For instance, in Supplementary Figure 4b it takes just ~ 35 removals to dismantle the original instance of the Moreno crime network, while the LCC size of the rewired networks after the same number of removals is still very large (i.e., $\sim 95\%$). This result confirms our insight. That is, existing topological correlations are learned and, consequently, exploited by the machine.

Dismantling results

In the main paper we compare our approach with the state-of-the-art algorithms. In Supplementary Table 2 we report the same results in numerical form.

The table also includes other commonly used static attack approaches that remove the nodes in descending importance order according to some node centrality metric. While many heuristics fall in this category, we compare with the removal of nodes in descending degree (*5*), betweenness (*5*) and PageRank (*6*). Our approach outperforms all these static approaches with a significant margin, even the ones with higher computational complexity (e.g., the betweenness-based one).

Dismantling of large networks

In Supplementary Table 3 we report the results on empirical complex large systems in numerical form.

[3]The configuration model of a network keeps the observed connectivity distribution while destroying topological correlations, meaning that feature correlations are lost.

In Supplementary Table 4 we also report the prediction (if any) and dismantling time of each of the methods to give a better idea on what their different computational complexities mean and translate into.

Dismantling curves

In Supplementary Figure 5, we display the dismantling of most of our test networks and compare with the state-of-the-art algorithms and with the heuristics introduced in the previous paragraph. As previously mentioned, one of the advantages of our approach is that we can choose the best model to reach a given objective. As an example, we show the models that lower the area under the curve (GDM AUC) and the removals number (GDM #Removals), which may overlap for some networks. We also show the dismantling performing the reinsertion phase and compare with state-of-the-art algorithms in Supplementary Figure 6.

Supplementary Note 5: Explaining the models

In this section we report the full results of the explanation experiments.

In order to compute the explanation sub-graphs, we extend the Pytorch Geometric's implementation of GNNExplainer to support regression tasks by using a Mean Square Error (MSE) loss function. We train the GNNExplainer (*7*) model for 600 epochs with a 0.01 learning rate, and mask out the edges with weight w lower than $E[w] + 1.5 \cdot \sigma_w$.

We show the explanation sub-graphs of the first four removed nodes for the corruption network in Supplementary Figure 7, while we report the features' weight (learned by GNNExplainer itself) for 21 of our test networks in Supplementary Figure 8.

For all the 45 real-world test networks, we also show the Articulation Points trend in Supplementary Figure 9 and the relation between the total number of APs in the network and the number of APs in the removal list (R) in Supplementary Figure 10.

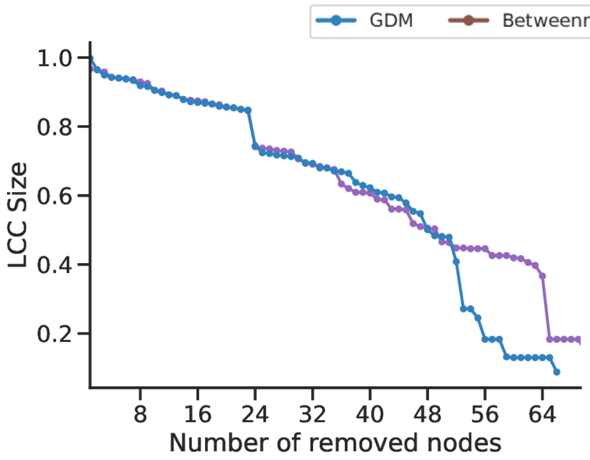

(a) arenas-meta degree

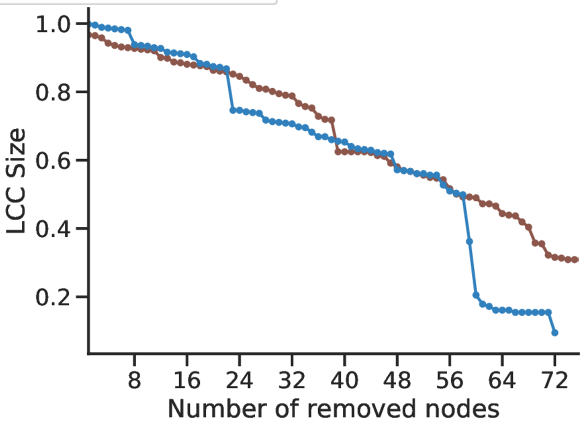

(b) arenas-meta betweenness

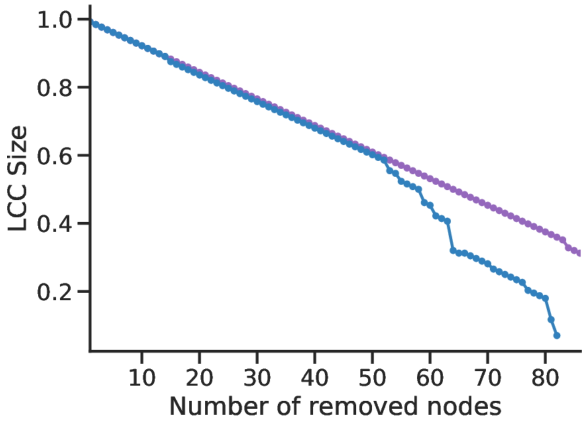

(c) foodweb-baywet degree

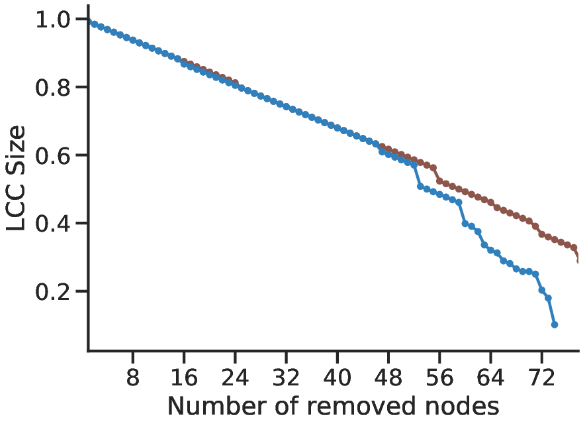

(d) foodweb-baywet betweenness

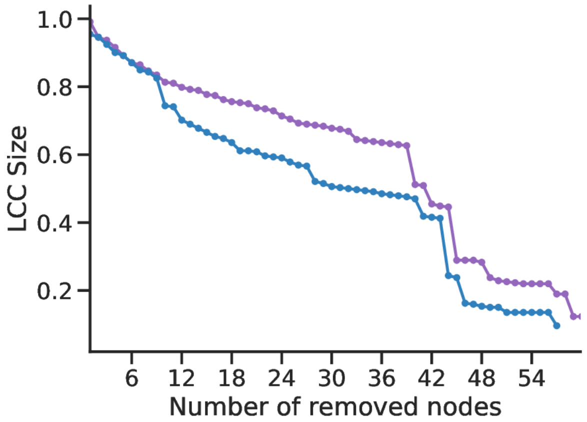

(e) inf-USAir97 degree

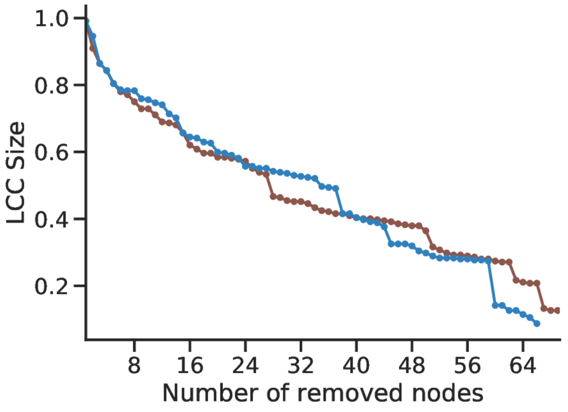

(f) inf-USAir97 betweenness

Supplementary Figure 3: Comparison of degree and betweenness vanilla heuristics with their GDM-enhanced versions on the arenas-meta, foodweb-baywet and inf-USAir97 networks.

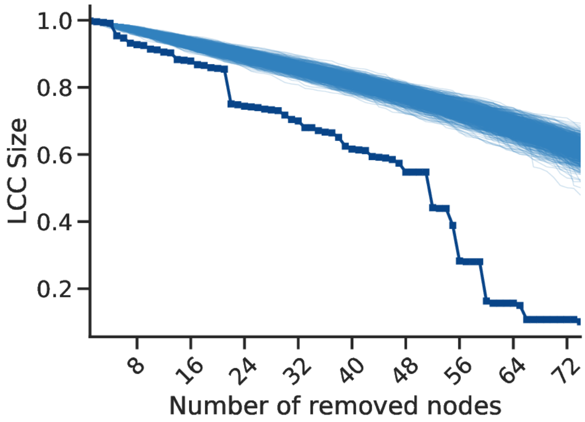

(a) arenas-meta network

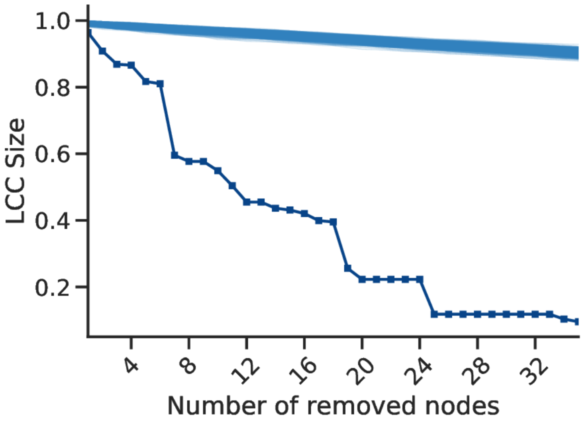

(b) Moreno crime network

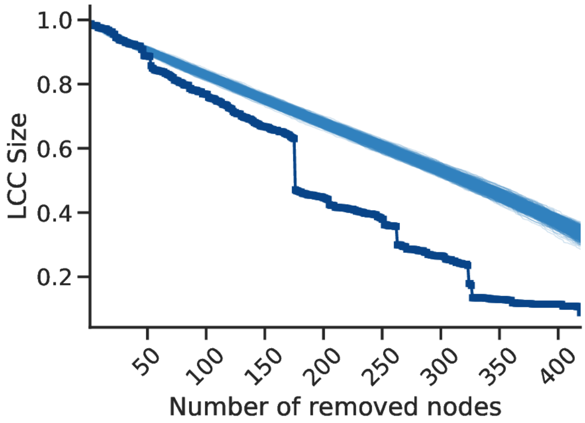

(c) opsahl-openflights network

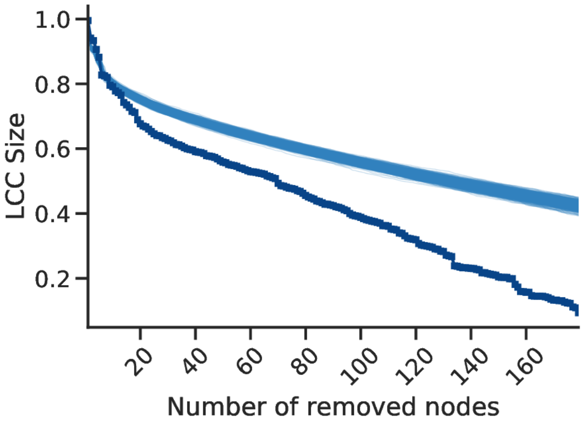

(d) route-views network

Supplementary Figure 4: Dismantling of original networks (dark blue) and 1000 configuration model rewirings for each (light blue).

Heuristic<br>Network	GDM	GND	EGND	Adaptive degree	EI σ_1	Pagerank	Degree	Betweenness	MS	EI σ_2	GDM +R	GND +R	CoreHD	MS +R	CI $\ell-2$
ARK201012_LCC	100.0	99.7	100.1	103.3	128.4	103.1	104.9	123.3	130.9	3883.7	94.5	87.6	92.6	95.8	114.6
advogato	100.0	108.0	105.5	101.8	111.6	150.1	113.4	114.8	112.6	494.5	94.8	97.5	102.1	102.7	98.8
arenas-meta	100.0	129.0	141.9	103.6	120.4	114.8	116.4	142.4	120.5	579.3	90.8	92.5	95.5	94.9	96.9
cfinder-google	100.0	160.4	246.5	99.5	233.7	113.5	141.3	377.9	682.8	1609.3	67.5	105.6	101.0	166.9	114.0
corruption	100.0	99.3	126.9	157.3	236.3	147.5	400.1	166.7	864.8	1141.9	97.6	147.4	138.6	139.6	176.6
dblp-cite	100.0	113.3	121.7	113.5	111.7	114.7	131.6	119.0	139.8	533.5	103.9	108.5	132.3	132.5	117.1
dimacs10-celegansneural	100.0	85.0	95.9	103.1	105.7	116.4	120.8	125.1	117.5	182.2	94.2	103.8	111.6	110.3	99.7
dimacs10-polblogs	100.0	107.5	97.1	102.1	115.5	112.5	117.9	114.8	107.5	262.3	98.4	108.4	106.0	104.9	104.6
econ-wm1	100.0	130.3	114.4	109.8	128.0	131.0	129.4	132.7	107.7	309.3	99.6	109.4	106.0	105.9	126.3
ego-twitter	100.0	116.8	115.8	108.9	103.0	107.8	108.8	133.3	167.3	6017.4	98.8	98.2	114.4	111.7	103.9
eu-powergrid	100.0	75.9	89.1	138.8	73.8	180.1	163.5	174.5	290.9	3313.0	64.4	66.5	83.4	92.8	109.4
foodweb-baydry	100.0	104.5	99.5	98.1	103.0	120.5	122.3	109.4	104.4	125.2	97.8	98.0	101.2	99.3	110.6
foodweb-baywet	100.0	110.2	108.4	99.6	103.9	123.6	125.4	112.9	106.8	128.3	98.5	108.5	102.1	101.8	113.0
inf-USAir97	100.0	112.4	117.8	130.4	147.0	117.1	139.1	128.6	164.0	633.6	100.1	117.2	103.7	107.6	129.8
internet-topology	100.0	95.6	95.8	99.1	113.9	109.2	131.4	122.9	138.6	3879.9	94.8	84.7	100.2	101.7	103.0
librec-ciaodvd-trust	100.0	113.1	115.5	117.6	129.4	120.5	139.8	114.9	126.6	634.5	104.3	114.4	124.4	126.3	126.1
librec-filmtrust-trust	100.0	108.9	118.3	117.7	112.8	131.8	148.4	158.9	168.7	1308.2	89.7	95.5	106.8	98.6	98.0
linux	100.0	97.9	101.1	116.2	84.5	176.0	190.8	365.1	150.0	1035.2	78.3	71.4	74.1	80.1	92.1
loc-brightkite	100.0	100.2	100.3	98.6	97.7	104.3	110.9	122.1	106.7	593.9	89.5	99.7	92.1	92.4	93.0
maayan-Stelzl	100.0	144.1	133.0	102.5	114.3	113.4	127.7	137.0	111.7	1269.6	96.3	113.4	107.1	105.2	105.4
maayan-figeys	100.0	104.3	120.2	100.7	155.9	127.3	146.9	153.4	129.5	1656.6	98.0	100.1	123.7	123.4	99.5
maayan-foodweb	100.0	111.5	94.6	114.7	147.8	118.9	123.8	126.2	154.6	268.7	100.0	125.5	136.1	144.4	173.9
maayan-vidal	100.0	111.0	106.7	103.3	101.6	109.1	110.6	123.9	114.1	843.9	90.1	102.5	95.6	97.9	97.3
moreno_crime_projected	100.0	105.8	86.0	191.2	139.2	157.6	218.8	180.6	976.7	2103.3	82.7	88.8	100.3	104.1	126.2
moreno_propro	100.0	115.9	123.6	115.6	87.9	126.1	123.5	146.7	145.2	1985.3	90.7	94.6	92.2	93.1	96.3
moreno_train	100.0	104.9	104.9	107.1	124.0	149.5	156.0	134.7	176.9	408.8	100.0	109.7	115.6	120.3	211.6
munmun_digg_reply_LCC	100.0	116.3	108.6	98.5	109.4	106.5	108.3	117.5	98.9	556.8	95.6	104.0	99.0	98.4	98.5
opsahl-openflights	100.0	101.2	106.2	127.2	109.9	123.2	135.4	123.6	157.3	807.7	84.4	92.0	102.6	111.3	120.9
opsahl-powergrid	100.0	36.9	69.4	148.6	37.0	173.4	180.9	183.9	164.3	1508.1	43.1	42.1	51.4	52.5	65.6
opsahl-ucsocial	100.0	122.1	116.1	99.9	118.5	105.9	109.9	109.8	108.8	342.0	97.0	106.1	105.8	106.0	101.7
oregon2_010526	100.0	106.8	101.5	108.8	131.1	101.6	130.5	114.6	162.0	3247.5	90.0	80.5	113.0	112.8	95.1
p2p-Gnutella06	100.0	128.5	120.4	108.5	108.6	111.6	125.1	118.4	108.7	274.0	101.4	120.4	110.1	108.4	109.1
p2p-Gnutella31	100.0	133.6	NaN	109.1	112.7	110.3	123.1	129.5	109.2	474.4	102.3	121.6	110.4	108.8	109.8
pajek-erdos	100.0	112.2	107.5	103.3	119.9	103.3	104.6	106.7	122.8	2790.7	98.2	106.9	116.7	113.9	101.0
petster-hamster	100.0	92.5	90.9	122.7	103.8	135.1	127.2	123.8	166.7	402.6	91.5	93.3	96.2	96.5	98.6
power-eris1176	100.0	199.1	218.0	340.2	171.7	253.5	622.5	430.2	632.6	1957.4	86.6	154.8	161.3	157.8	153.7
route-views	100.0	99.3	99.1	101.8	133.2	103.5	103.5	112.3	131.5	4340.9	94.0	82.0	93.0	95.2	112.5
slashdot-threads	100.0	100.1	102.2	99.5	122.5	104.6	105.4	114.2	117.6	1495.8	96.1	95.8	115.7	115.1	97.9
slashdot-zoo	100.0	99.2	100.1	95.6	120.9	103.3	106.8	124.0	112.4	683.8	95.2	97.7	106.9	105.9	96.5
subelj_jdk	100.0	107.6	110.2	115.1	113.0	144.2	181.5	346.9	144.6	1275.7	80.9	84.7	84.8	81.0	103.4
subelj_jung-j	100.0	102.1	111.6	122.0	118.4	150.7	185.7	334.6	143.1	1295.0	80.1	88.5	82.9	72.2	101.5
web-EPA	100.0	148.4	157.6	102.2	141.1	104.9	109.7	137.7	158.1	1471.9	101.1	115.6	133.8	132.8	107.3
web-webbase-2001	100.0	127.6	130.0	165.0	196.6	216.3	165.4	207.7	3603.1	55066.4	64.7	50.1	76.6	82.6	80.9
wikipedia_link_kn	100.0	107.3	102.6	103.7	113.2	124.8	143.6	140.3	128.8	NaN	92.9	98.0	113.9	113.5	96.8
wikipedia_link_li	100.0	120.3	145.4	132.8	151.8	120.5	165.2	110.6	211.9	1049.4	107.2	151.0	177.5	174.5	157.8
Average	100.0	111.7	115.4	119.1	123.6	128.7	151.1	158.9	273.3	2596.4	91.5	100.8	106.9	108.7	112.1

Supplementary Table 2: Per-method area under the curve (AUC) of real-world networks dismantling. The lower the better. The dismantling target for each method is 10% of the network size. We compute the AUC value by integrating the $LCC(x)/|N|$ values using Simpson's rule, and each value is scaled to the one of our approach (GDM) for the same network. +R means that the reinsertion phase is performed. CoreHD and CI are compared to other +R algorithms as they include the reinsertion phase. EGND for p2p-Gnutella31 is missing as the computation was killed after 10d.

Heuristic<br>Network	GDM	GND	MS	GDM +R	GND +R	MS +R	CoreHD
citeseer	100.0	102.2	111.2	92.8	91.3	95.0	94.3
com-dblp	100.0	109.6	184.5	91.5	108.3	92.4	91.2
digg-friends	100.0	100.9	140.5	97.0	103.6	120.7	121.2
douban	100.0	120.8	132.7	102.6	129.3	131.6	132.9
email-EuAll	100.0	97.0	192.1	100.0	100.0	147.4	148.5
hyves	100.0	109.3	133.6	101.6	109.6	131.9	133.6
loc-gowalla	100.0	103.2	105.4	89.7	91.9	91.0	90.5
munmun_twitter_social	100.0	105.2	140.5	100.2	112.4	138.5	137.3
petster-catdog-household	100.0	100.7	164.7	95.4	98.0	143.4	144.7
tech-RL-caida	100.0	104.8	147.2	86.9	94.3	82.8	80.2
twitter_LCC	100.0	93.6	98.8	85.3	81.4	83.0	84.7
wordnet-words	100.0	120.4	234.5	100.0	110.8	111.0	109.7
Average	100.0	105.6	148.8	95.3	102.6	114.1	114.1

Supplementary Table 3: Per-method area under the curve (AUC) of real-world large networks dismantling. The lower the better. The dismantling target for each method is 10% of the network size. We compute the AUC value by integrating the $LCC(x)/|N|$ values using Simpson's rule, and each value is scaled to the one of our approach (GDM) for the same network. +R means that the reinsertion phase is performed. CoreHD and CI are compared to other +R algorithms as they include the reinsertion phase.

Heuristic<br>Network	Prediction time GDM	Prediction time CoreHD	Dismantle time GDM	Dismantle time GND	Dismantle time MS
citeseer	00:00:03.4	00:00:22.9	01:30:17.1	03:43:51.6	01:26:21.5
com-dblp	00:00:02.9	00:00:14.9	00:22:30.7	04:57:25.6	00:59:38.4
digg-friends	00:00:02.8	00:00:19.9	00:08:01.9	00:30:55.5	01:11:37.4
douban	00:00:01.3	00:00:06.1	00:01:10.1	00:03:34.8	00:11:40.4
email-EuAll	00:00:02.4	00:00:07.8	00:00:10.7	00:01:14.9	00:09:49.0
hyves	00:00:13.5	00:00:36.6	03:08:02.7	08:21:22.8	02:03:26.9
loc-gowalla	00:00:02.0	00:00:15.9	00:17:22.3	01:27:28.0	00:46:15.0
munmun_twitter_social	00:00:04.3	00:00:14.3	00:00:53.5	00:07:53.4	00:29:13.9
petster-catdog-household	00:00:03.9	00:00:40.6	00:44:20.5	03:58:17.1	02:16:02.8
tech-RL-caida	00:00:01.8	00:00:12.1	00:07:23.7	04:14:34.1	00:29:30.8
twitter_LCC	00:00:04.4	00:00:13.0	00:32:01.0	05:33:36.3	00:19:18.8
wordnet-words	00:00:01.4	00:00:12.1	00:03:34.0	01:23:52.1	00:22:28.5

Supplementary Table 4: Real-world large networks dismantling timings. The lower the better. Time format is HH:MM:SS.s. *MS* and *GND* do not have prediction time as they refresh the predictions during the dismantling, while there is no *CoreHD* dismantling column as we use our dismantler.

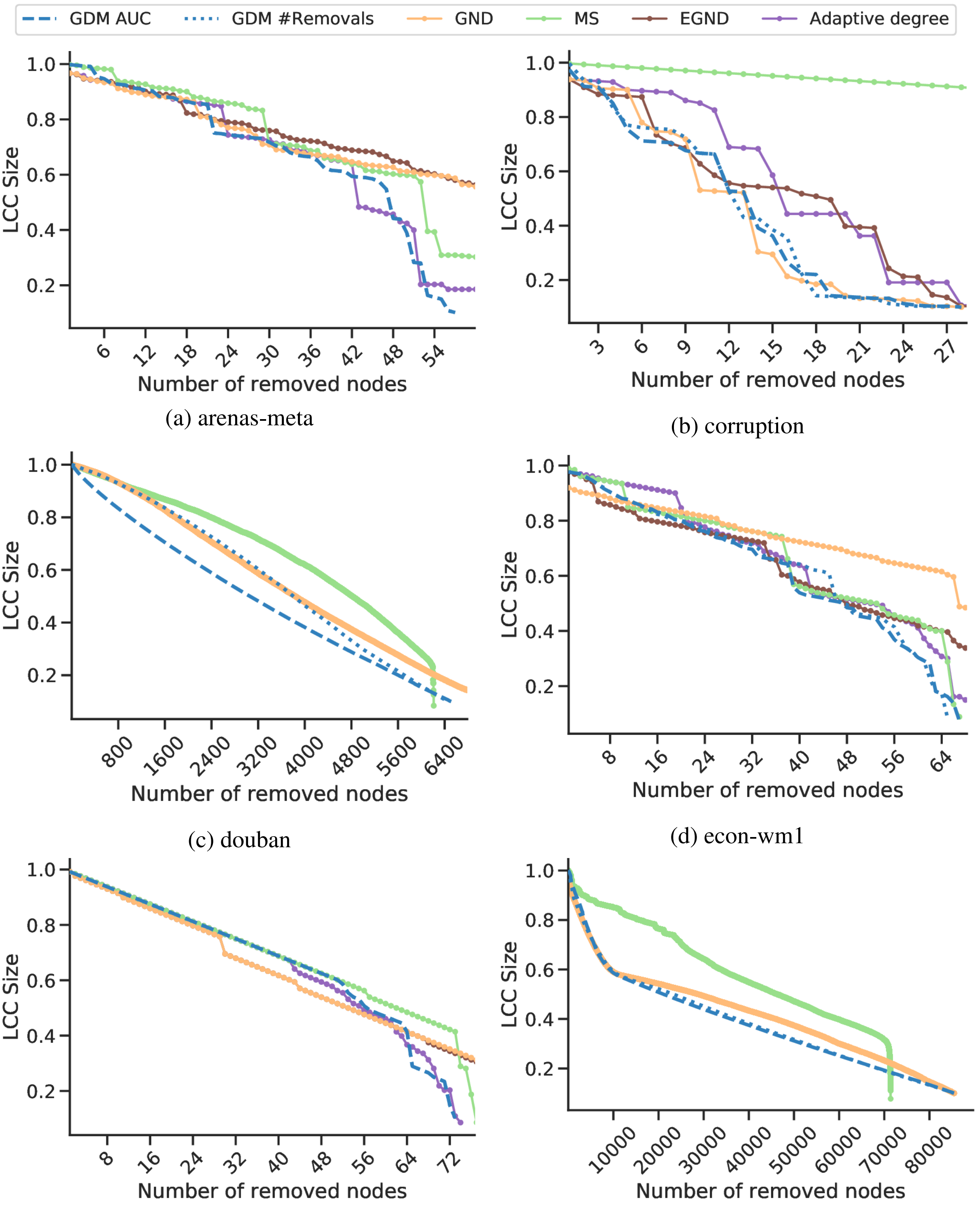

(a) arenas-meta

(b) corruption

(c) douban

(d) econ-wm1

(e) foodweb-baywet

(f) hyves

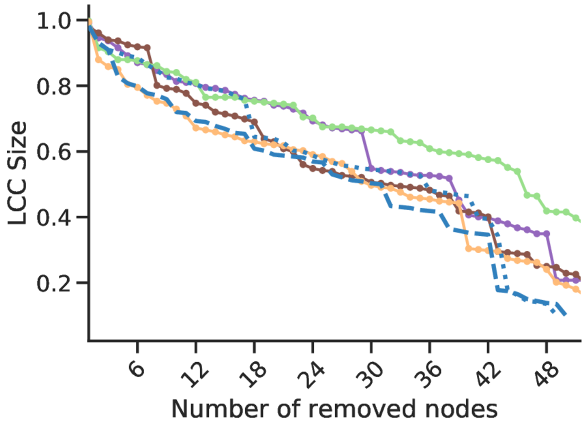

(g) inf-USAir97

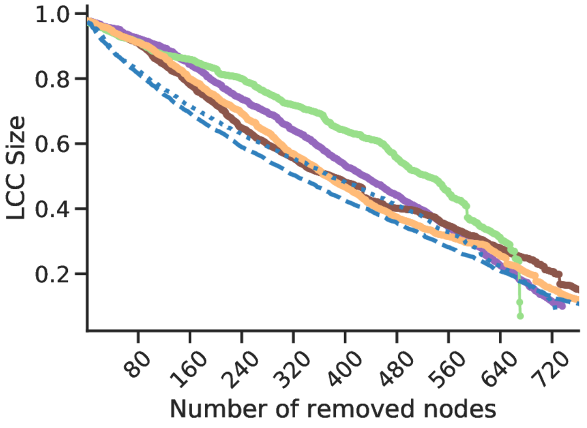

(h) librec-ciaodvd-trust

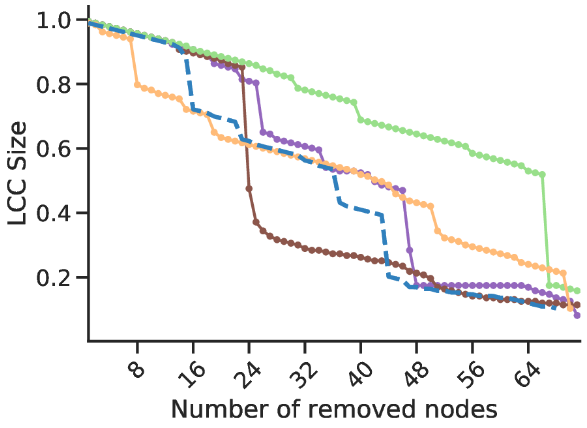

(i) maayan-foodweb

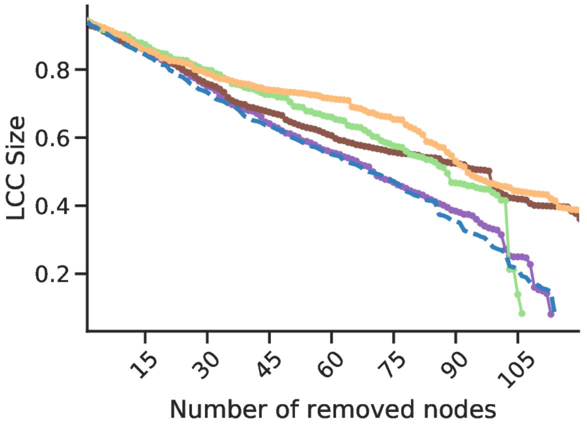

(j) maayan-Stelzl

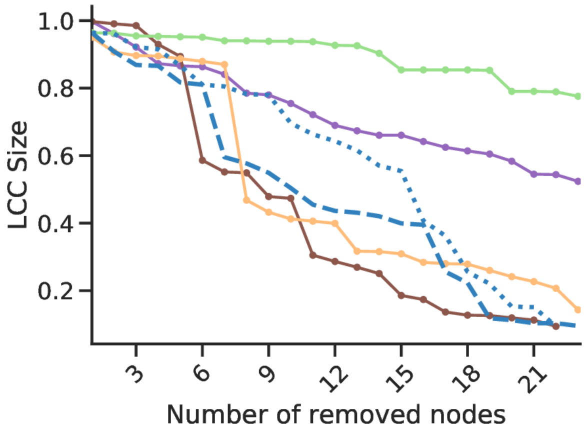

(k) moreno-crime-projected

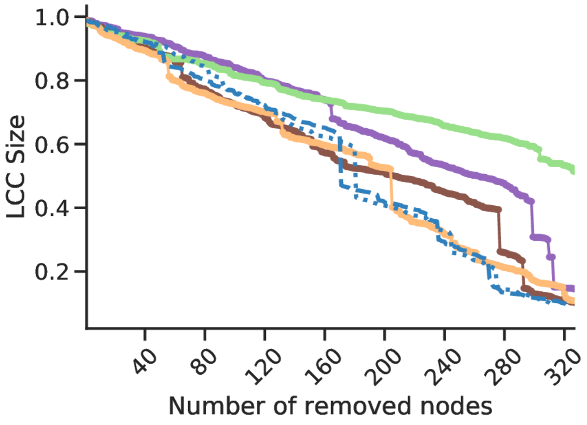

(l) opsahl-openflights

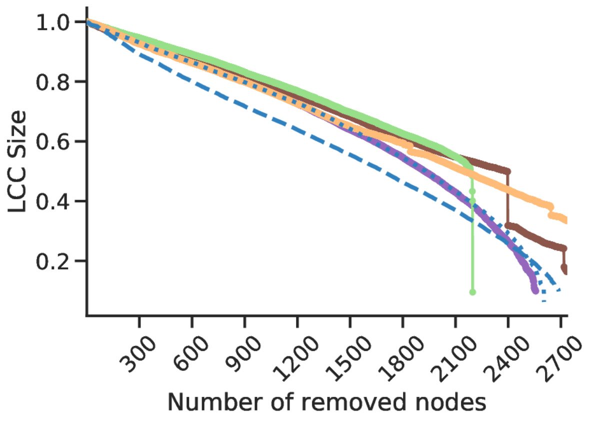

(m) p2p-Gnutella06

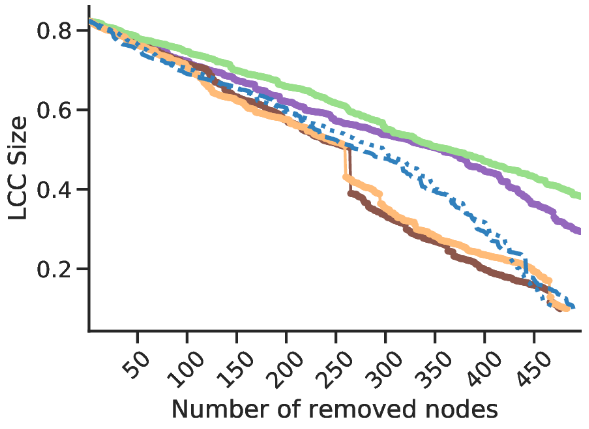

(n) petster-hamster

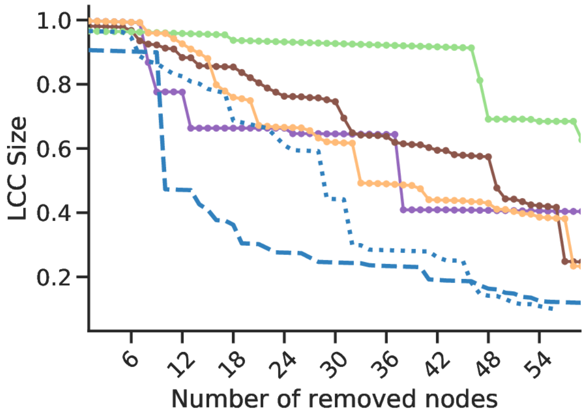

(o) power-eris1176

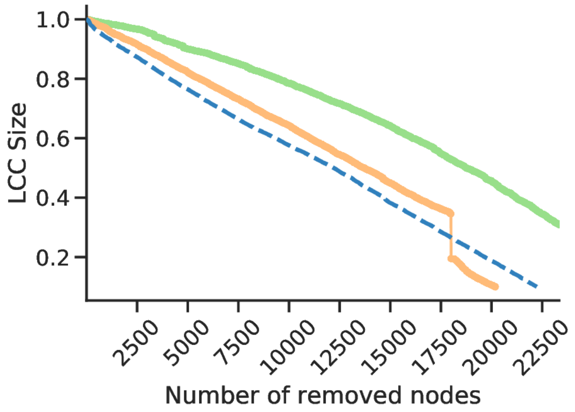

(p) tech-RL-caida

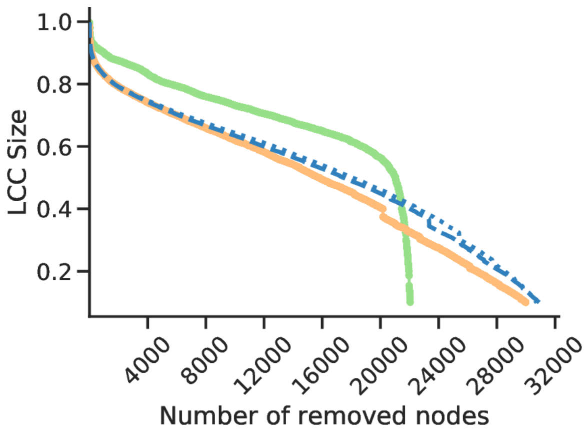

(q) twitter_LCC

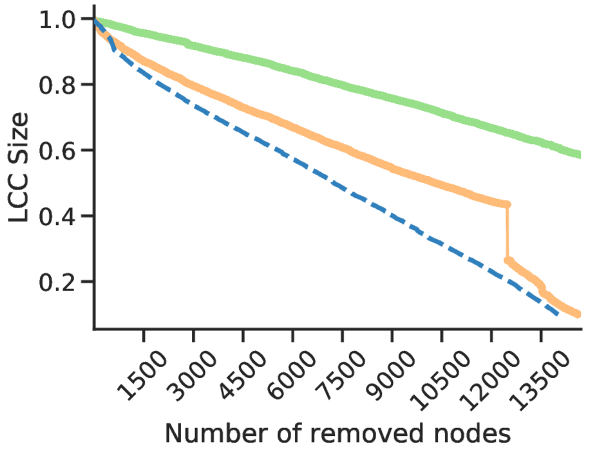

(r) wordnet-words

Supplementary Figure 5: Dismantling of some networks in our test set. We compare against the algorithms without reinsertion in Supplementary Table 2 and Supplementary Table 3 and show both the models with lower area under the curve (GDM AUC) and with lower number of removals (GDM #Removals), which may overlap for some networks.

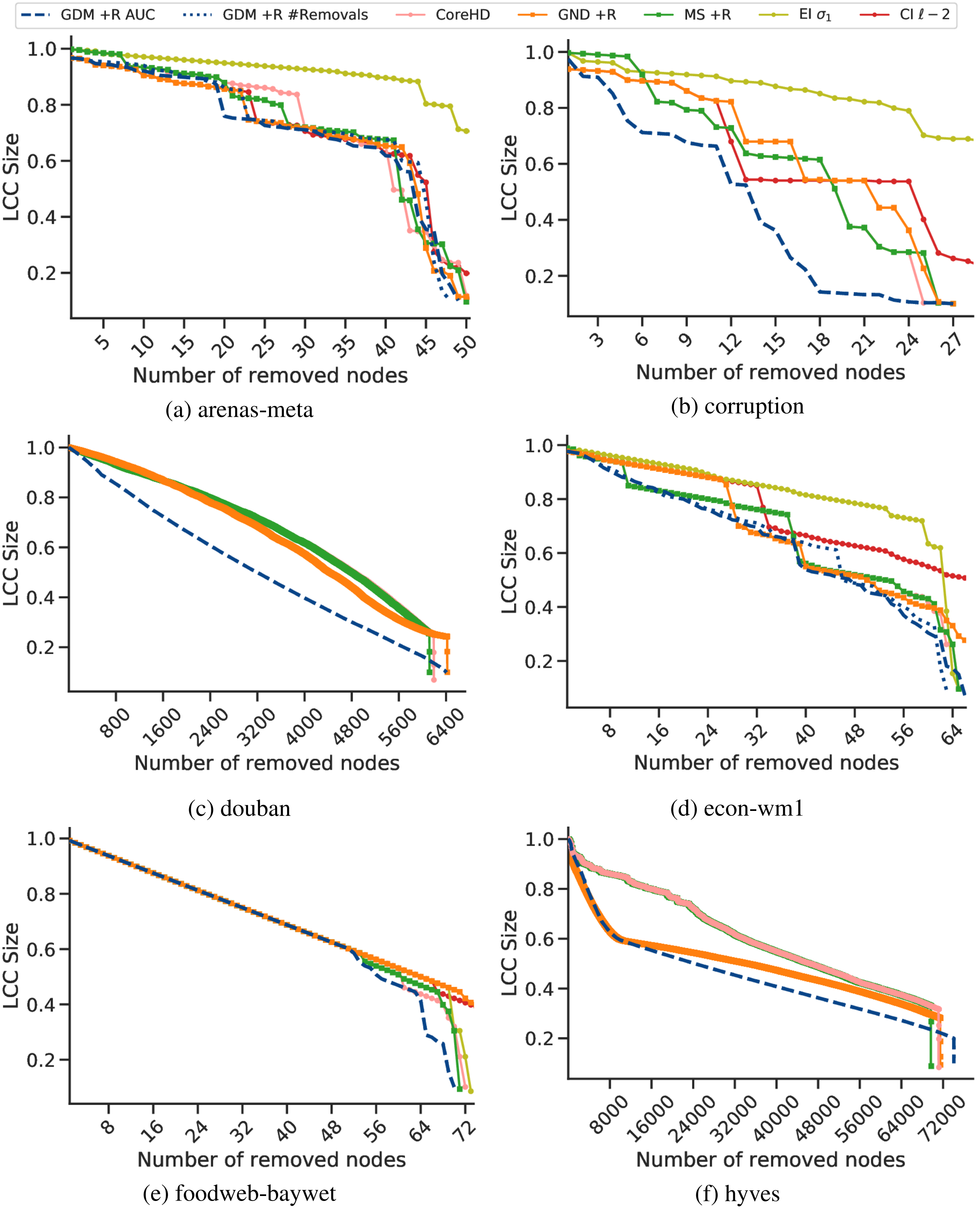

(a) arenas-meta

(b) corruption

(c) douban

(d) econ-wm1

(e) foodweb-baywet

(f) hyves

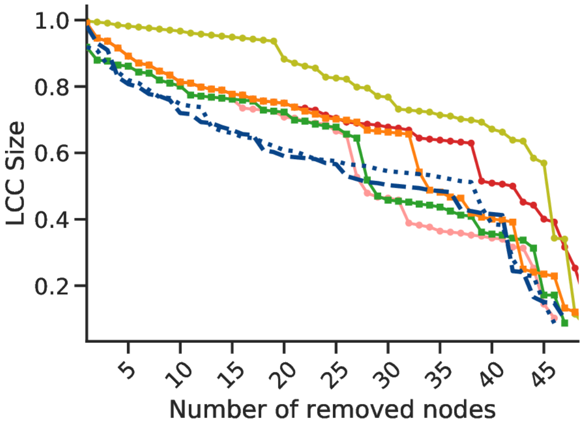

(g) inf-USAir97

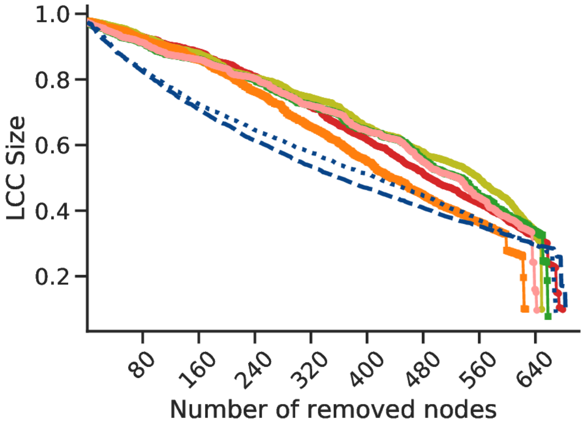

(h) librec-ciaodvd-trust

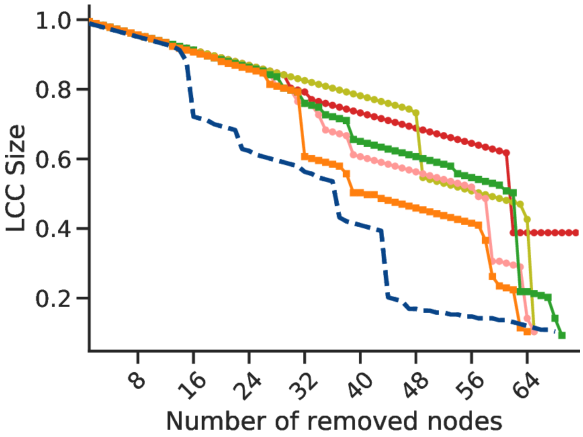

(i) maayan-foodweb

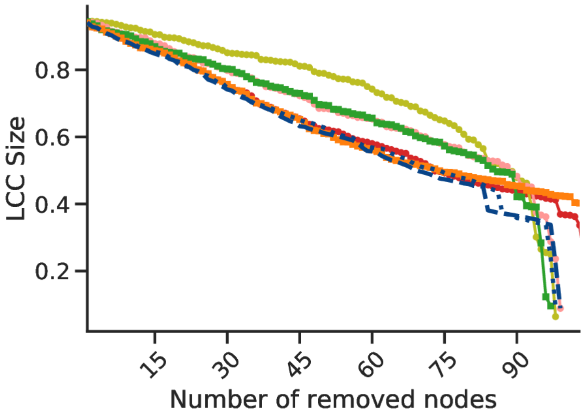

(j) maayan-Stelzl

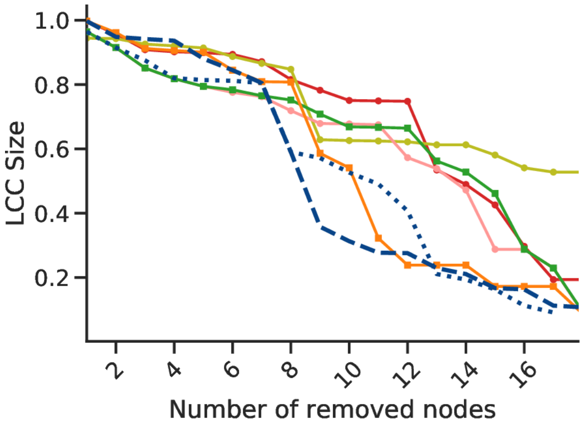

(k) moreno-crime-projected

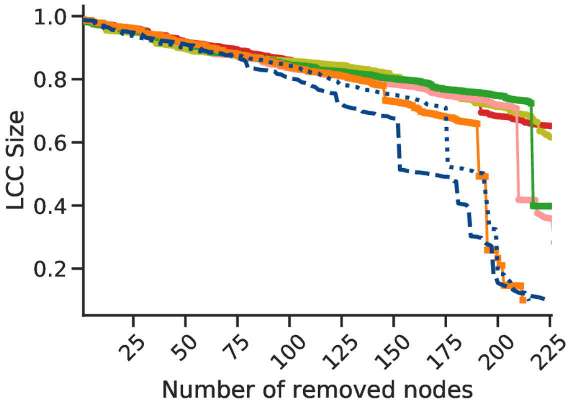

(l) opsahl-openflights

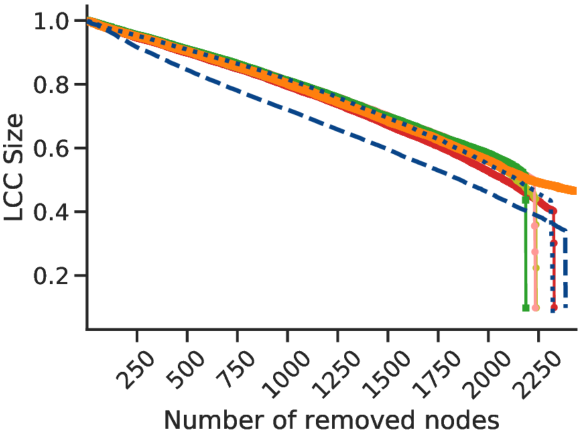

(m) p2p-Gnutella06

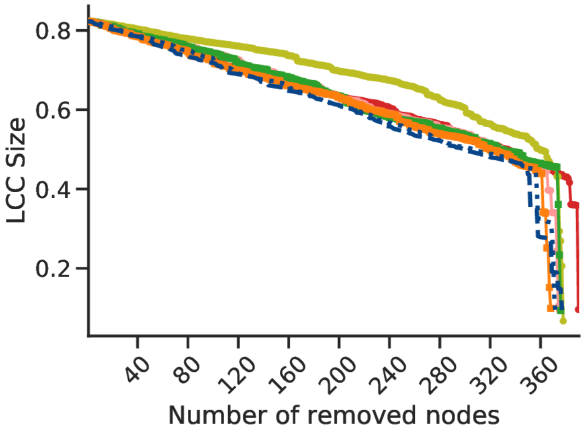

(n) petster-hamster

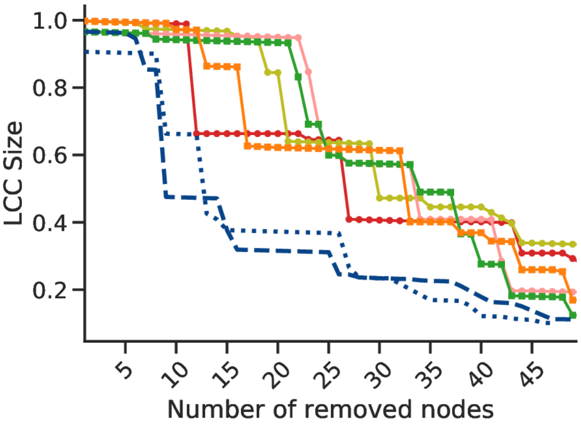

(o) power-eris1176

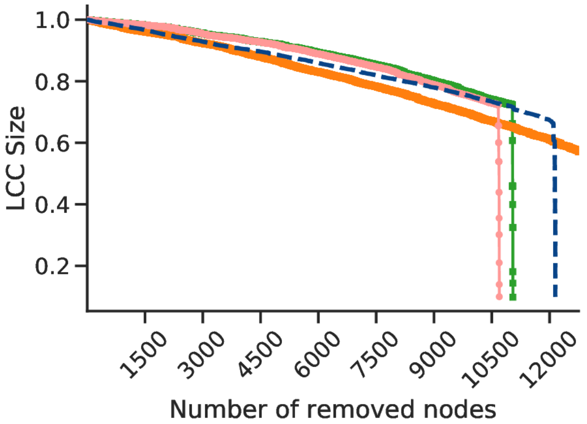

(p) tech-RL-caida

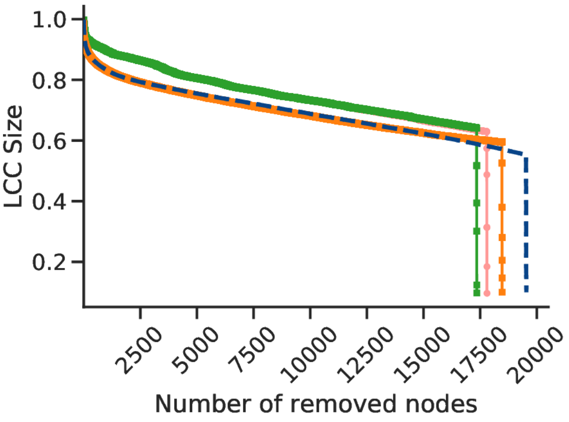

(q) twitter_LCC

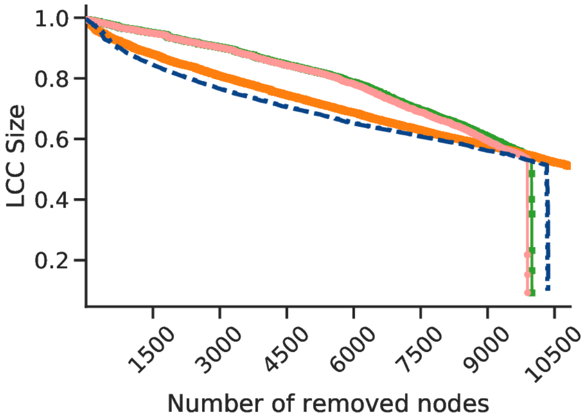

(r) wordnet-words

Supplementary Figure 6: Dismantling of some networks in our test set. We compare against the algorithms with reinsertion phase in Supplementary Table 2 and Supplementary Table 3 and show both the models with lower area under the curve (GDM +R AUC) and with lower number of removals (GDM +R #Removals), which may overlap for some networks.

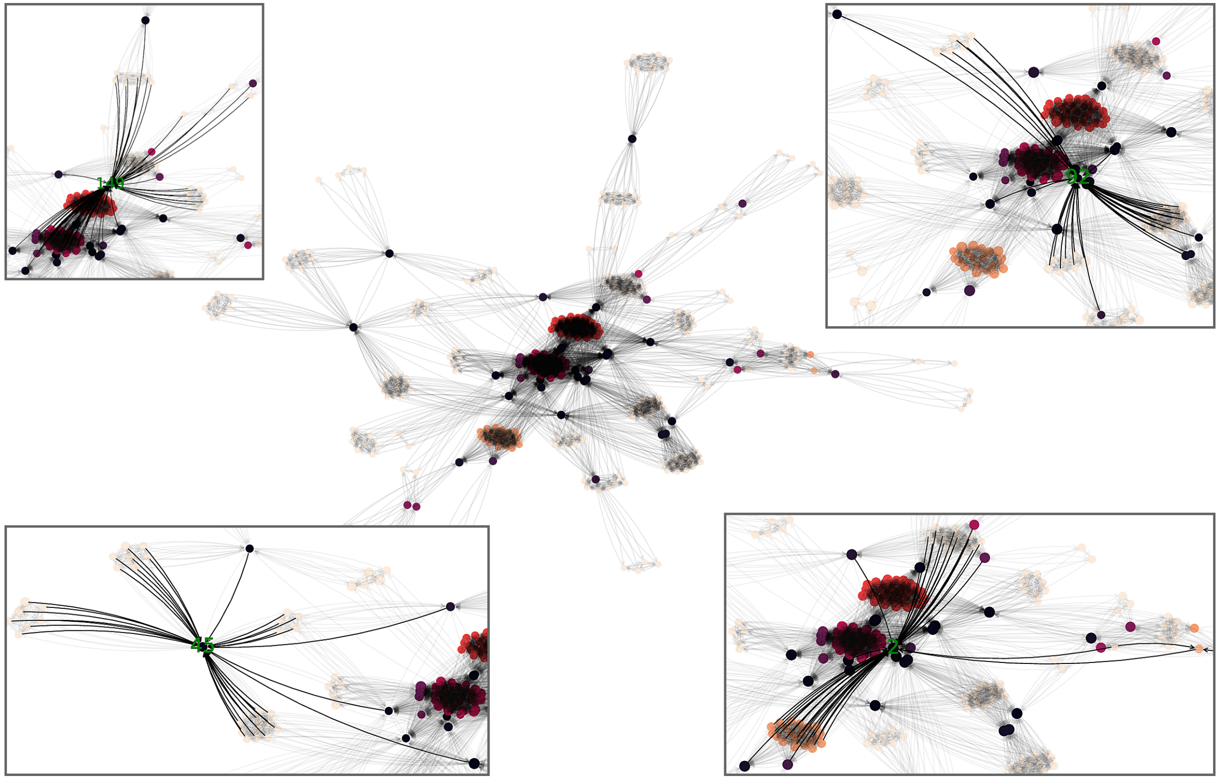

Supplementary Figure 7: Explanation sub-graphs for the first four nodes of the Brazilian corruption network. The model is targeting nodes that act as bridge between multiple clusters and the choice is also based on neighboring nodes that are bridges themselves.

Supplementary Note 6: More Early Warning Ω examples

In addition to the example applications of Ω illustrated in the main paper, we also test if it can detect the collapse of other systems. In particular, we show the SciKit European power-grid (eu-powergrid) under random failures, degree or Min-Sum + Reinsertion phase attacks in Supplementary Figure 11, and also various American roads under Generalized Network Dismantling + Reinsertion phase attacks in Supplementary Figure 12. In all these scenarios, Ω is able to detect the system damage and reaches warning levels before the system collapse actually happens, even in case of multiple large connected components detaching from the larger one as the attack goes on.

Supplementary Note 7: Dataset

In Supplementary Table 5 we list the test networks used in our experiments with their category and size (number of nodes and edges). Those networks model systems from various domains (e.g., biological, infrastructure and social data and so on), and range from a few hundred of nodes to more than one million. For more about each network, we refer the reader to the original source.

Supplementary Note 8: Test environment

Here we detail the environment where our experiments were performed and the tools used.

All experiments ran on a shared machine equipped with two Intel Xenon E5-2620 CPUs, 128GB RAM and a two core nVidia Tesla K80 (with 12GB VRAM each). More details about the drivers used and the full package dependency list of our code can be found in the code package.

Concerning the other algorithms used in our comparison (i.e., GND, EGND, MS, CoreHD

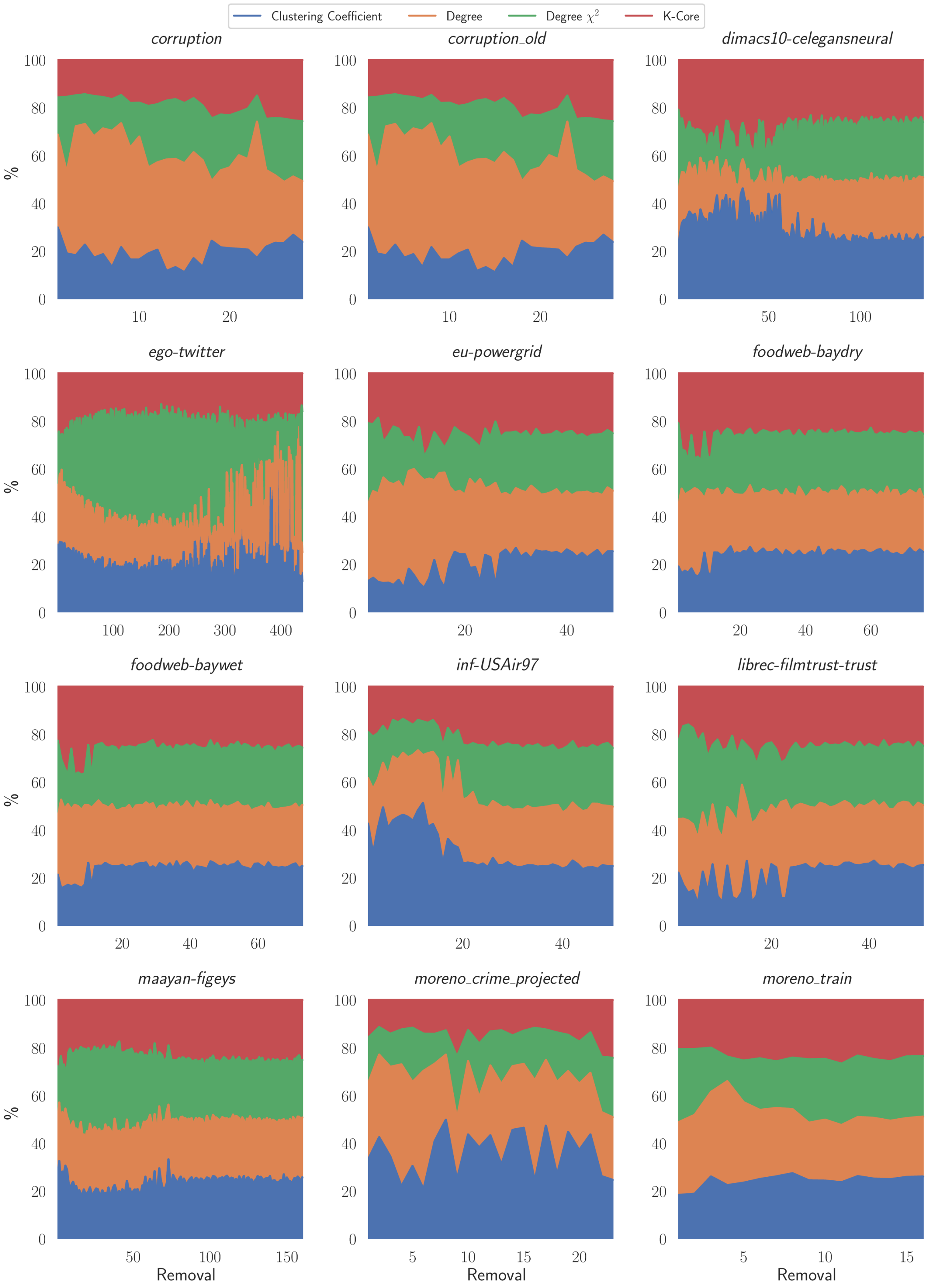
Clustering Coefficient
Degree
Degree χ^2
K-Core
corruption
corruption_old
dimacs10-celegansneural
ego-twitter
eu-powergrid
foodweb-baydry
foodweb-baywet
inf-USAir97
librec-filmtrust-trust
maayan-figeys
moreno_crime_projected
moreno_train
%
Removal

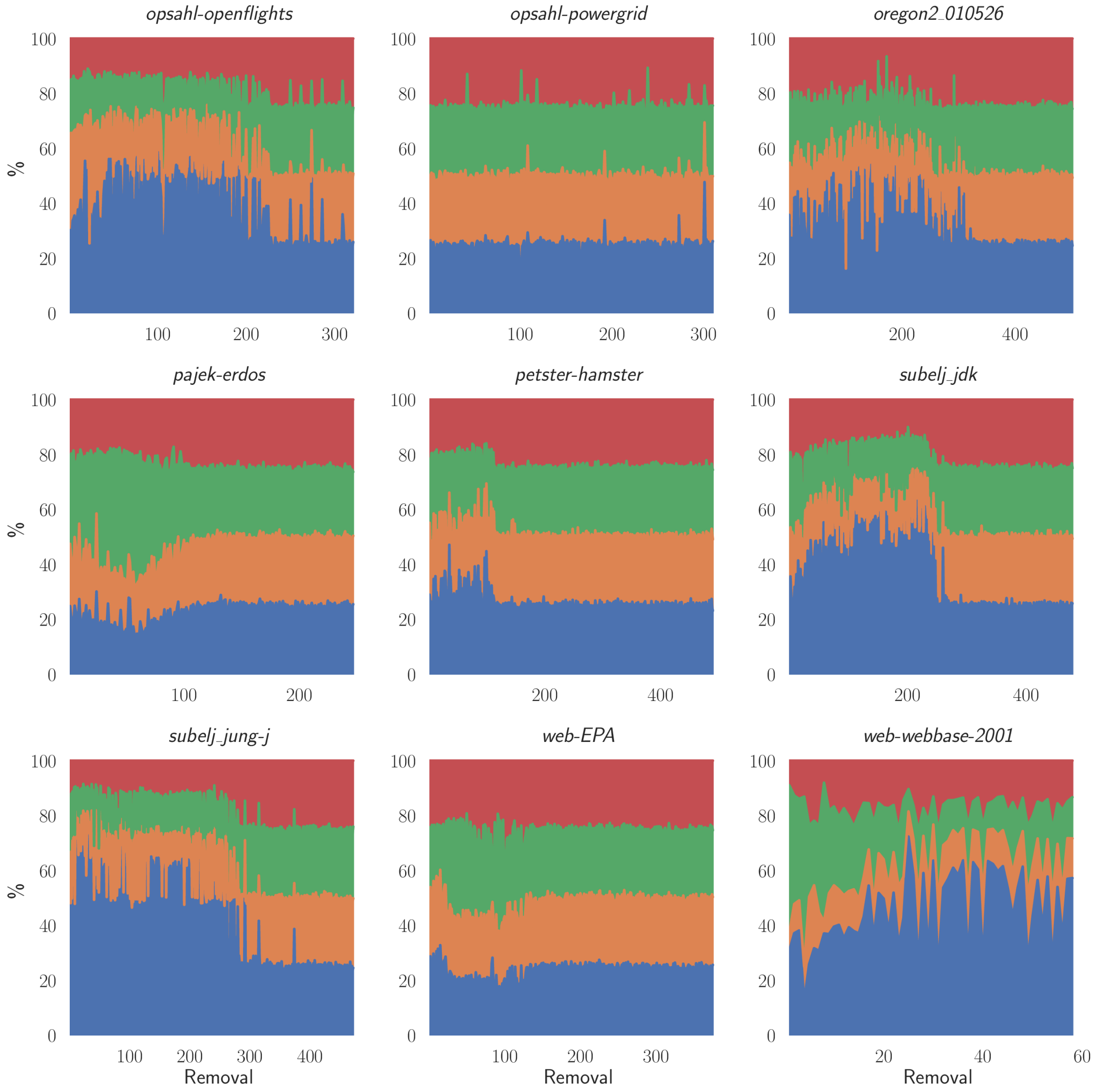

Supplementary Figure 8: Features' importance trend. Relative features' importance in the computation of each p_n value, provided by GNNExplainer, in removal order.

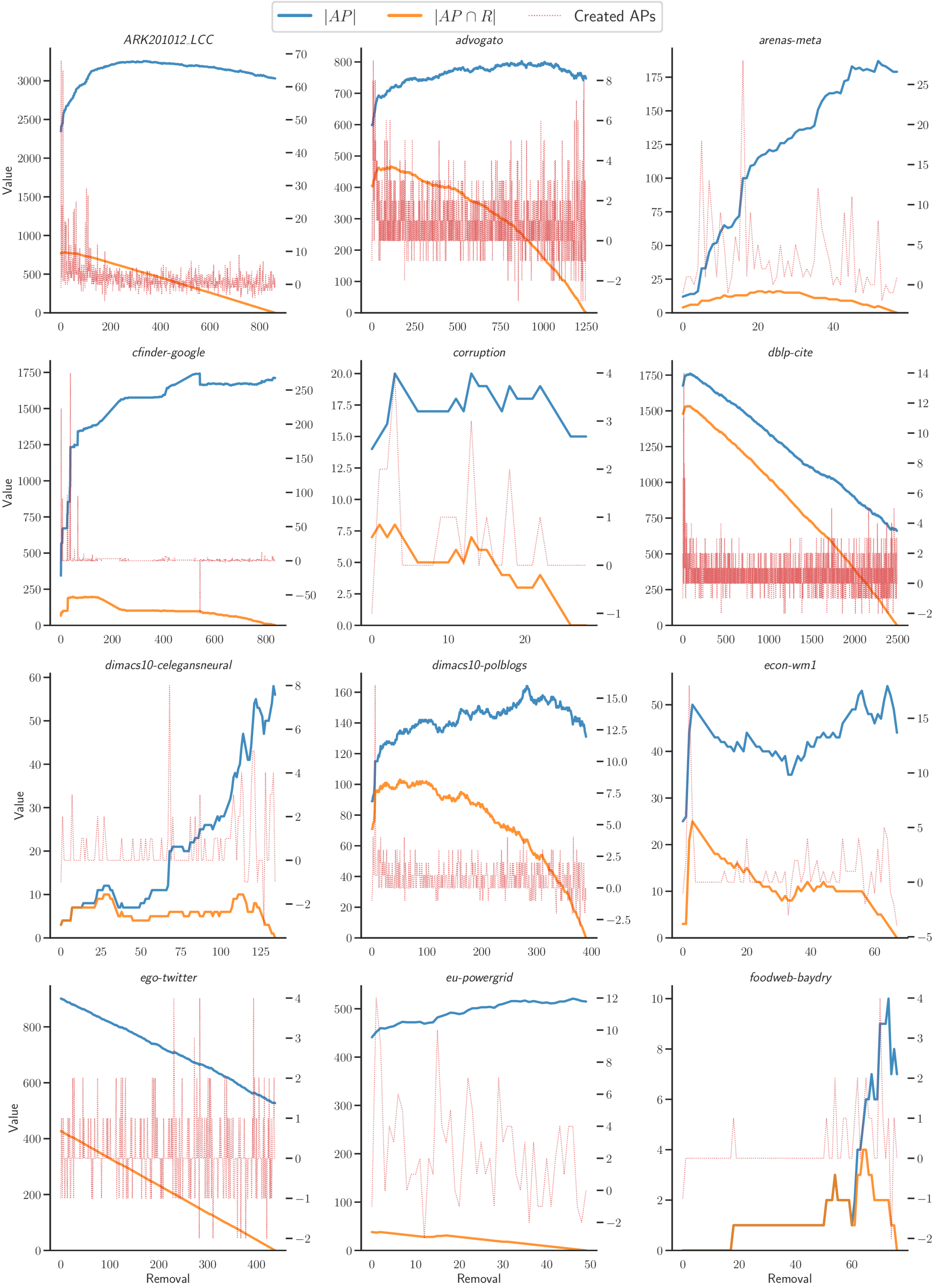
$|AP|$
$|AP \cap R|$
Created APs
ARK201012_LCC
advogato
arenas-meta
cfinder-google
corruption
dblp-cite
dimacs10-celegansneural
dimacs10-polblogs
econ-wm1
ego-twitter
eu-powergrid
foodweb-baydry
Value
Removal

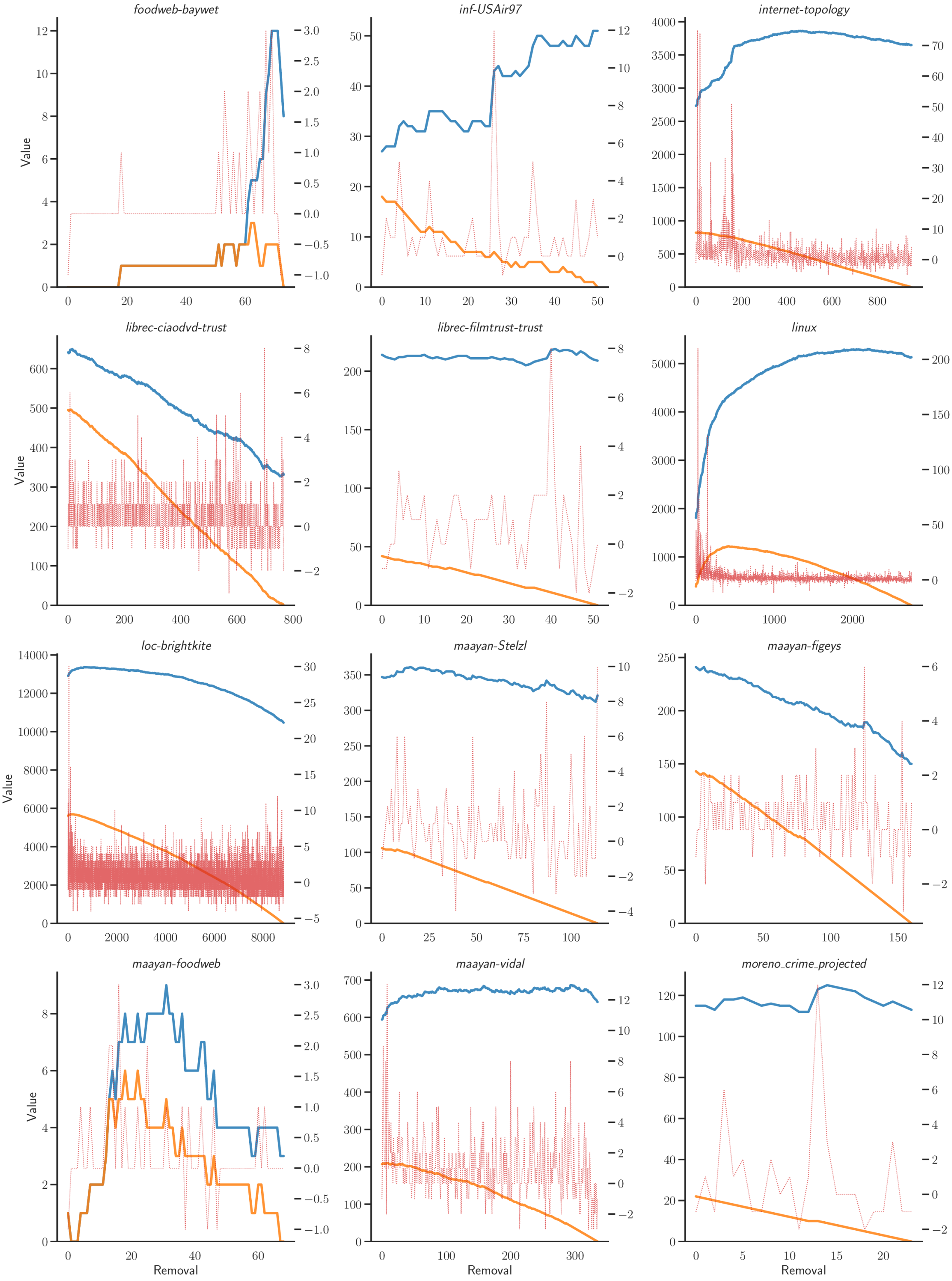
foodweb-baywet
inf-USAir97
internet-topology
librec-ciaodvd-trust
librec-filmtrust-trust
linux
loc-brightkite
maayan-Stelzl
maayan-figeys
maayan-foodweb
maayan-vidal
moreno_crime_projected
Value
Removal

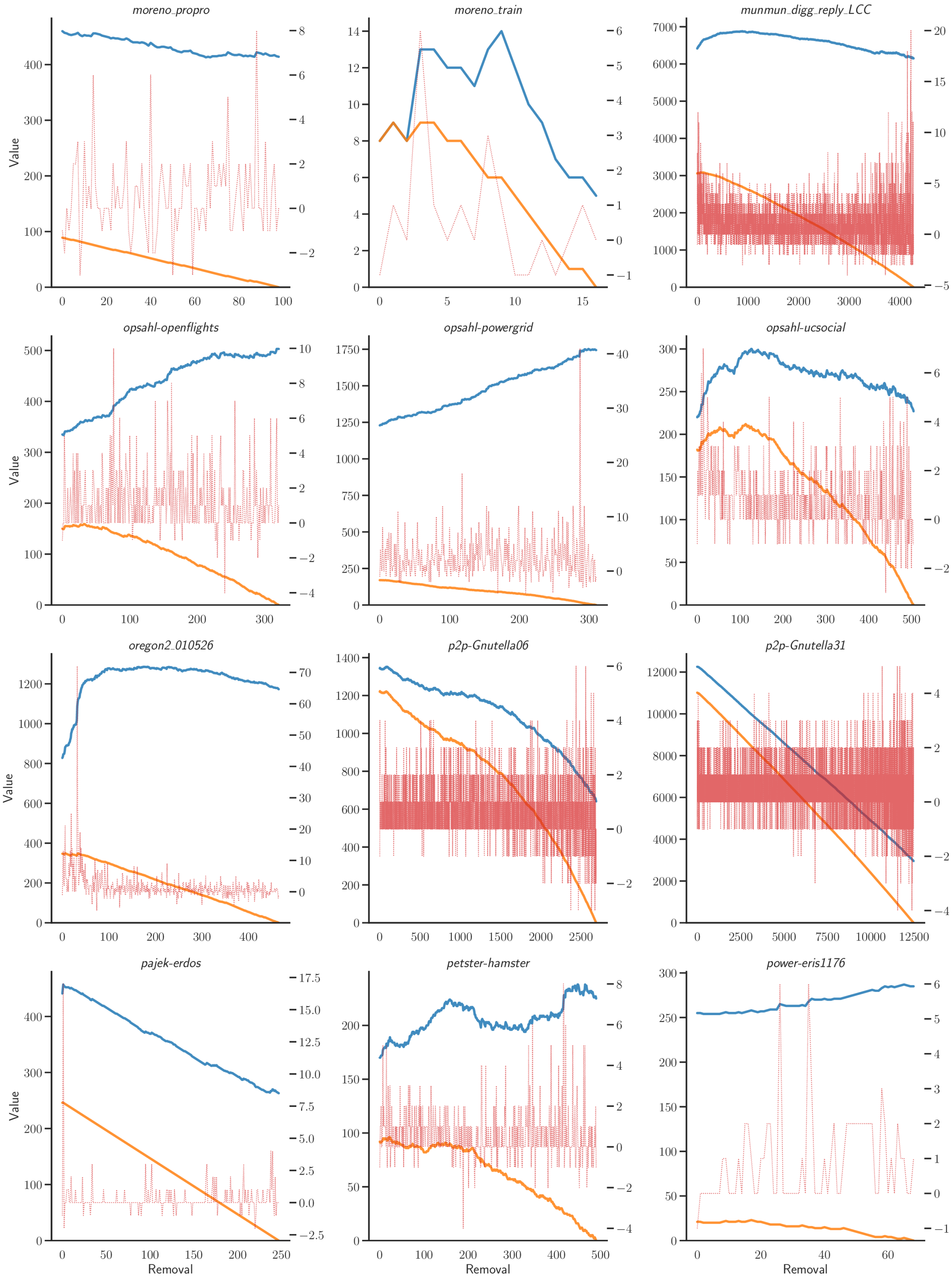
moreno_propro
moreno_train
munmun_digg_reply_LCC
opsahl-openflights
opsahl-powergrid
opsahl-ucsocial
oregon2_010526
p2p-Gnutella06
p2p-Gnutella31
pajek-erdos
petster-hamster
power-eris1176
Value
Removal

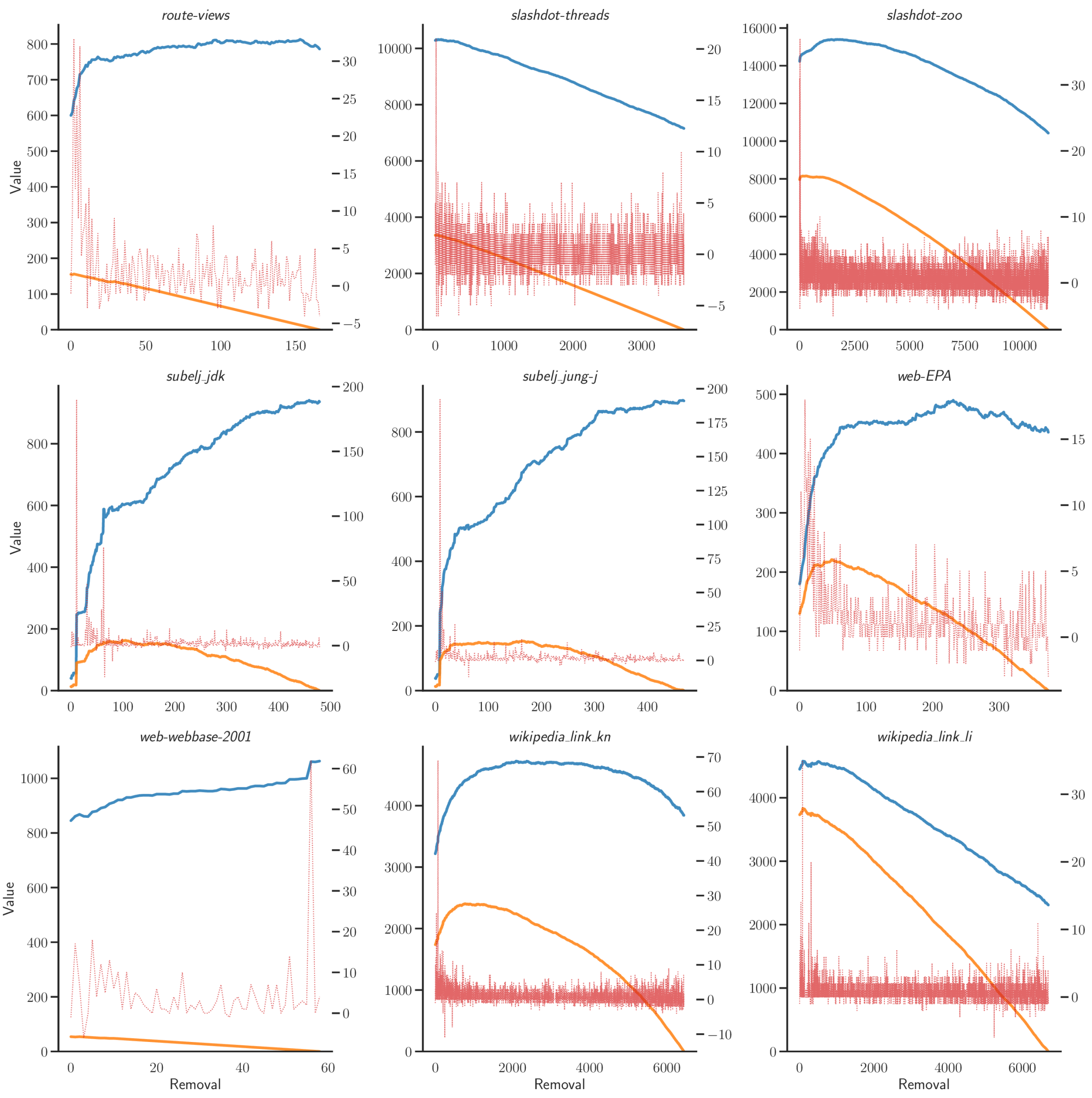

Supplementary Figure 9: Articulation Point trend. We compute, removal after removal, the number of APs in the network ($|AP|$), the number of APs in the removal list ($|AP \cap R|$) and the number of created APs.

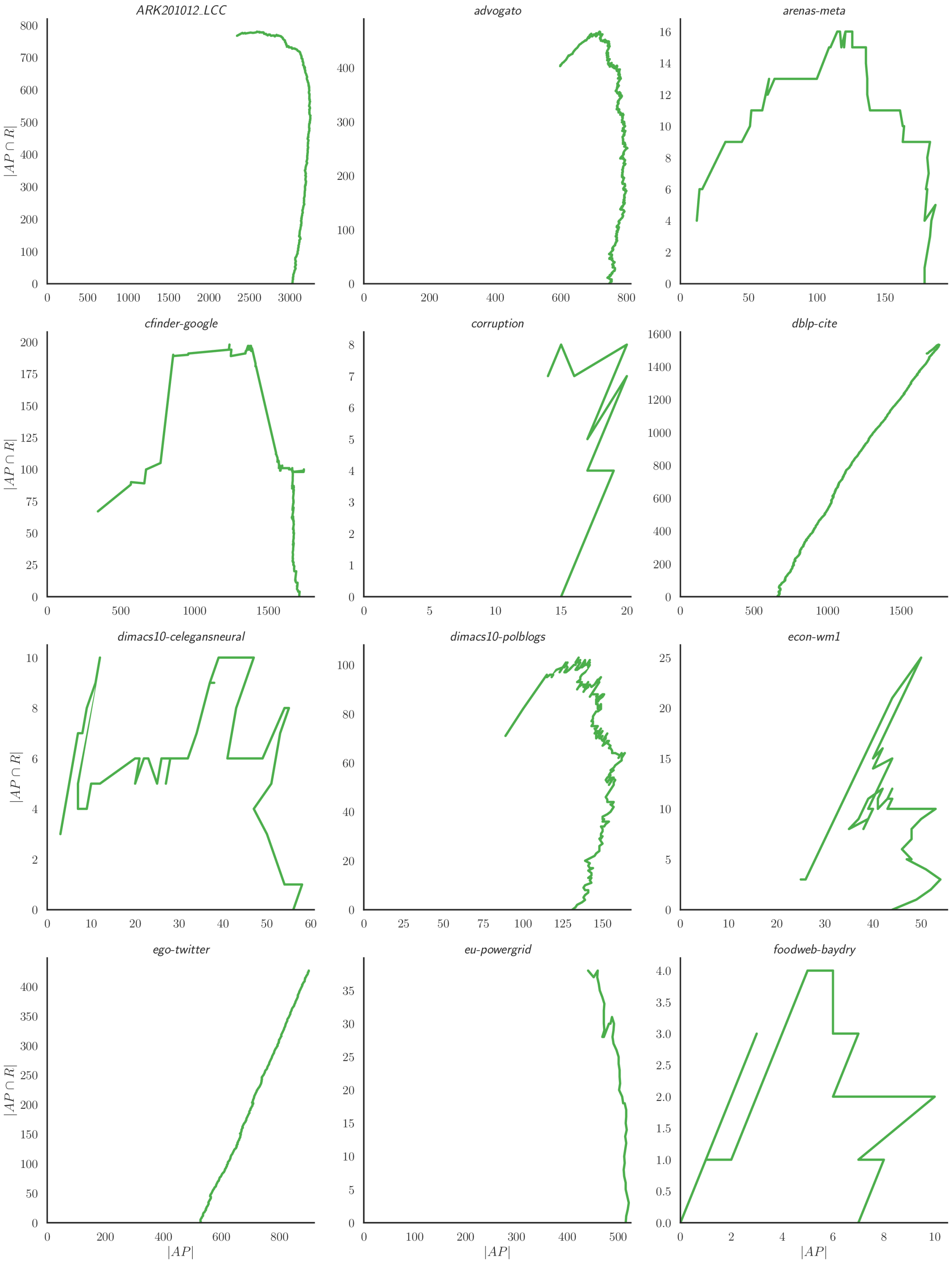
ARK201012_LCC
advogato
arenas-meta
cfinder-google
corruption
dblp-cite
dimacs10-celegansneural
dimacs10-polblogs
econ-wm1
ego-twitter
eu-powergrid
foodweb-baydry
$|AP \cap R|$
$|AP|$

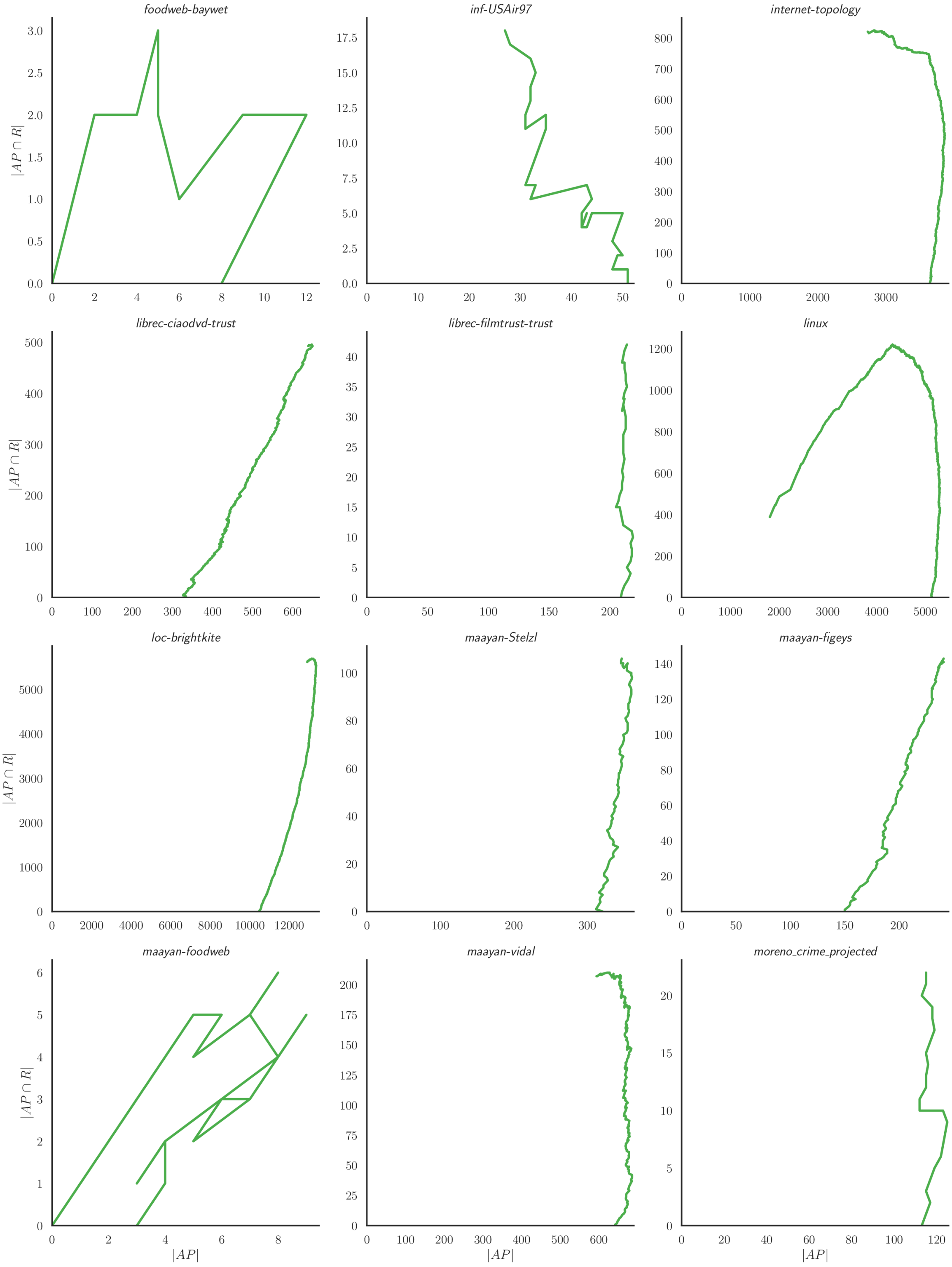
foodweb-baywet
inf-USAir97
internet-topology
librec-ciaodvd-trust
librec-filmtrust-trust
linux
loc-brightkite
maayan-Stelzl
maayan-figeys
maayan-foodweb
maayan-vidal
moreno_crime_projected
$|AP \cap R|$
$|AP|$

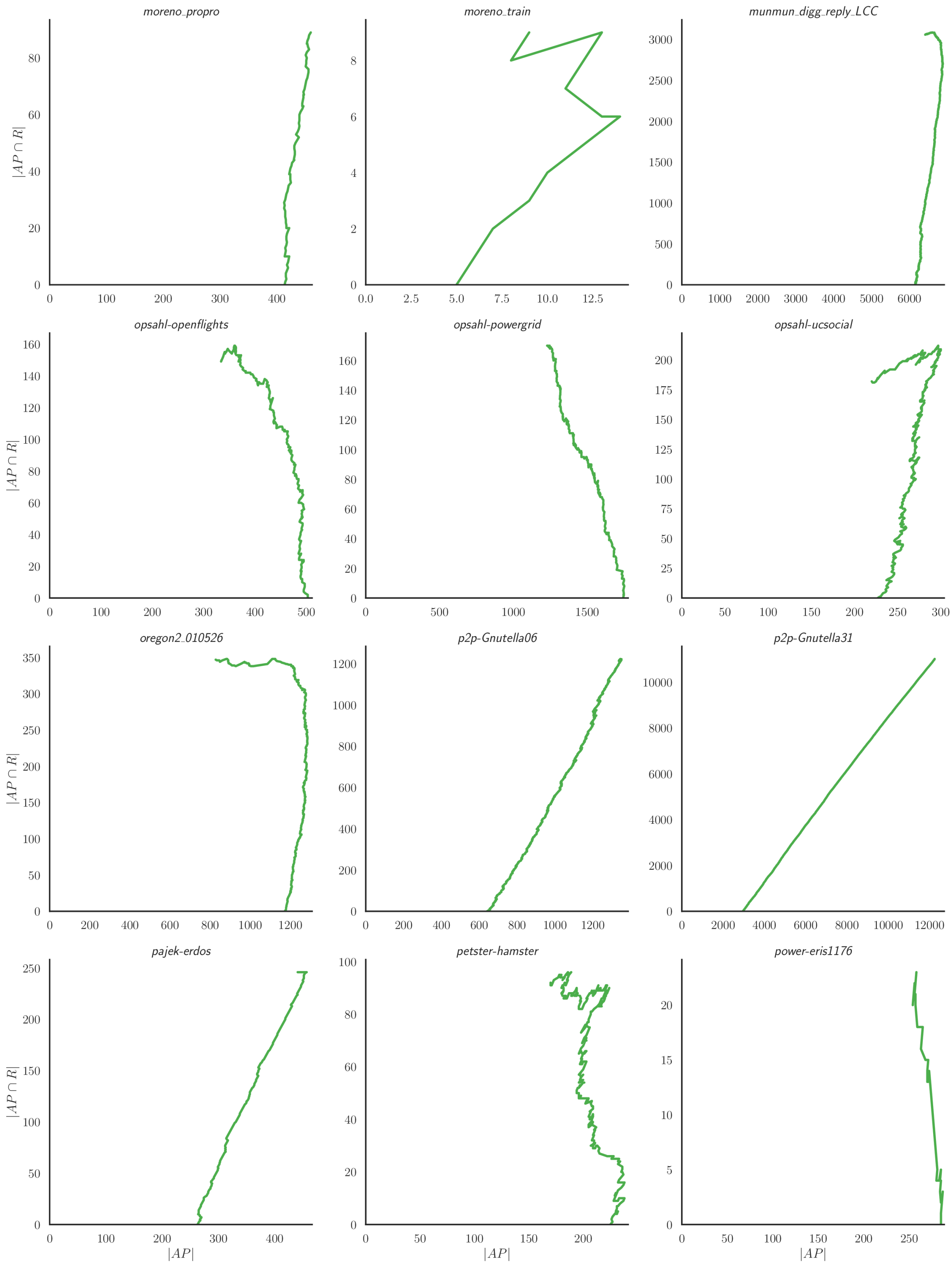
moreno_propro
moreno_train
munmun_digg_reply_LCC
opsahl-openflights
opsahl-powergrid
opsahl-ucsocial
oregon2_010526
p2p-Gnutella06
p2p-Gnutella31
pajek-erdos
petster-hamster
power-eris1176
$|AP \cap R|$
$|AP|$

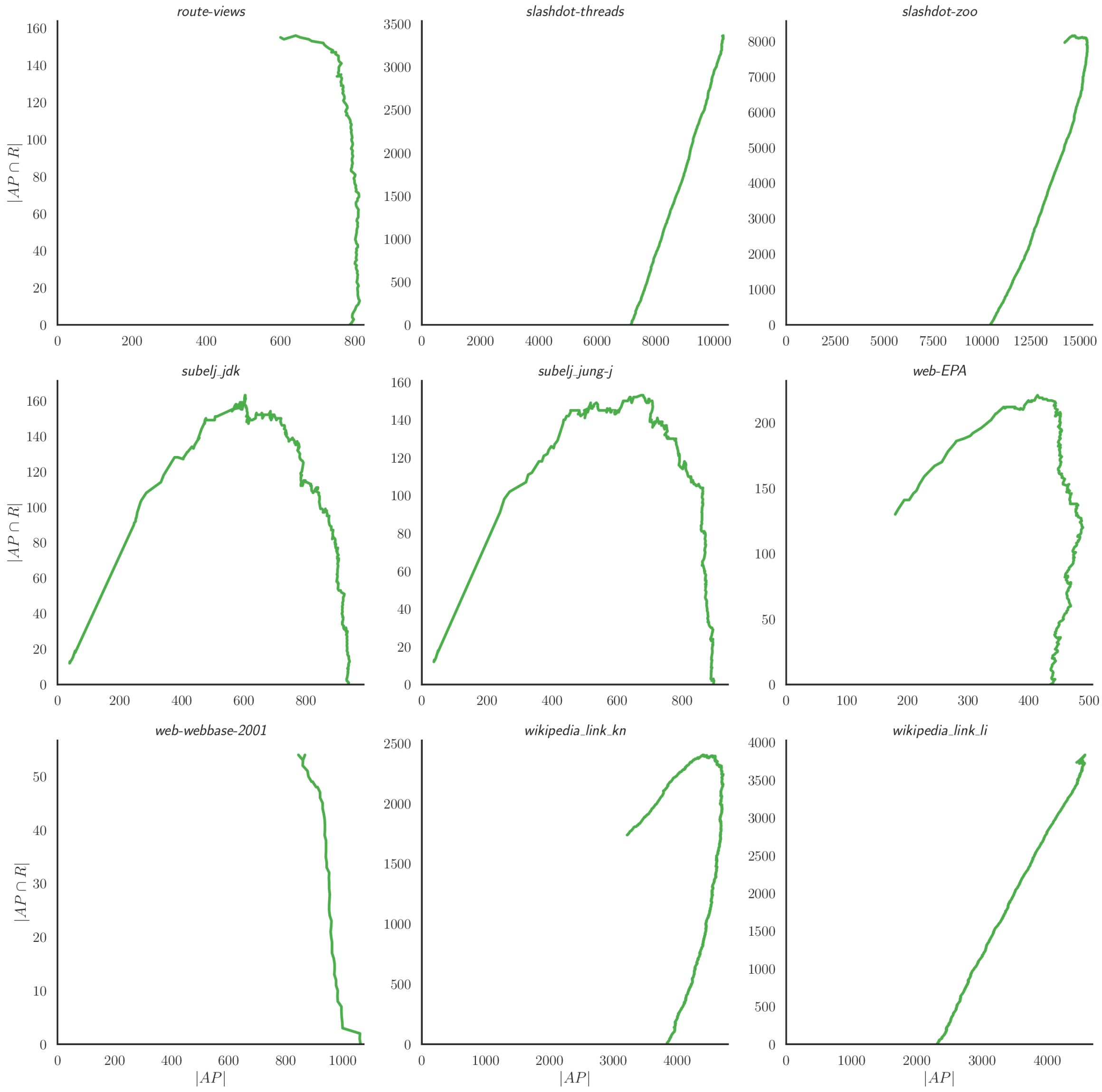

Supplementary Figure 10: Relation between the number of APs and the number of APs in the removal list. The two are related by a kind of deterministic dynamics, resembling the one which characterizes chaotic systems and, specifically, chaotic maps such as the logistic map or the Hénon map, where parabolic attractors emerge when the state of the system at the $n+1$-th step is plotted against the state at the n-th step. In our case, the n-th step coincides with the removal of the n-th node in the removal list. The shape of the resulting attractor provides a strong characterization of the system and its robustness.

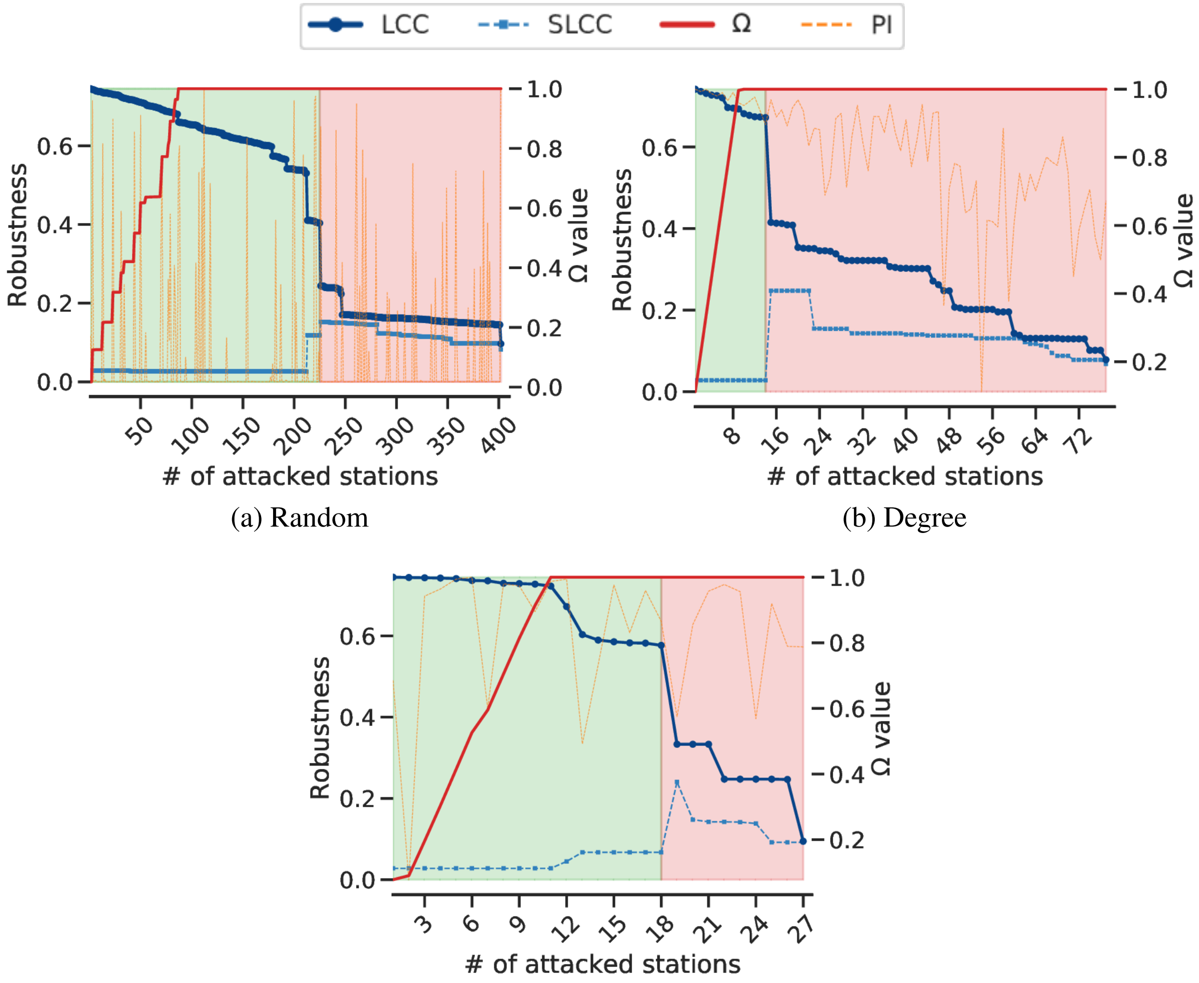

(a) Random

(b) Degree

(c) MS + R

Supplementary Figure 11: Early Warning values for the SciKit European powergrid under random failures and targeted attacks.

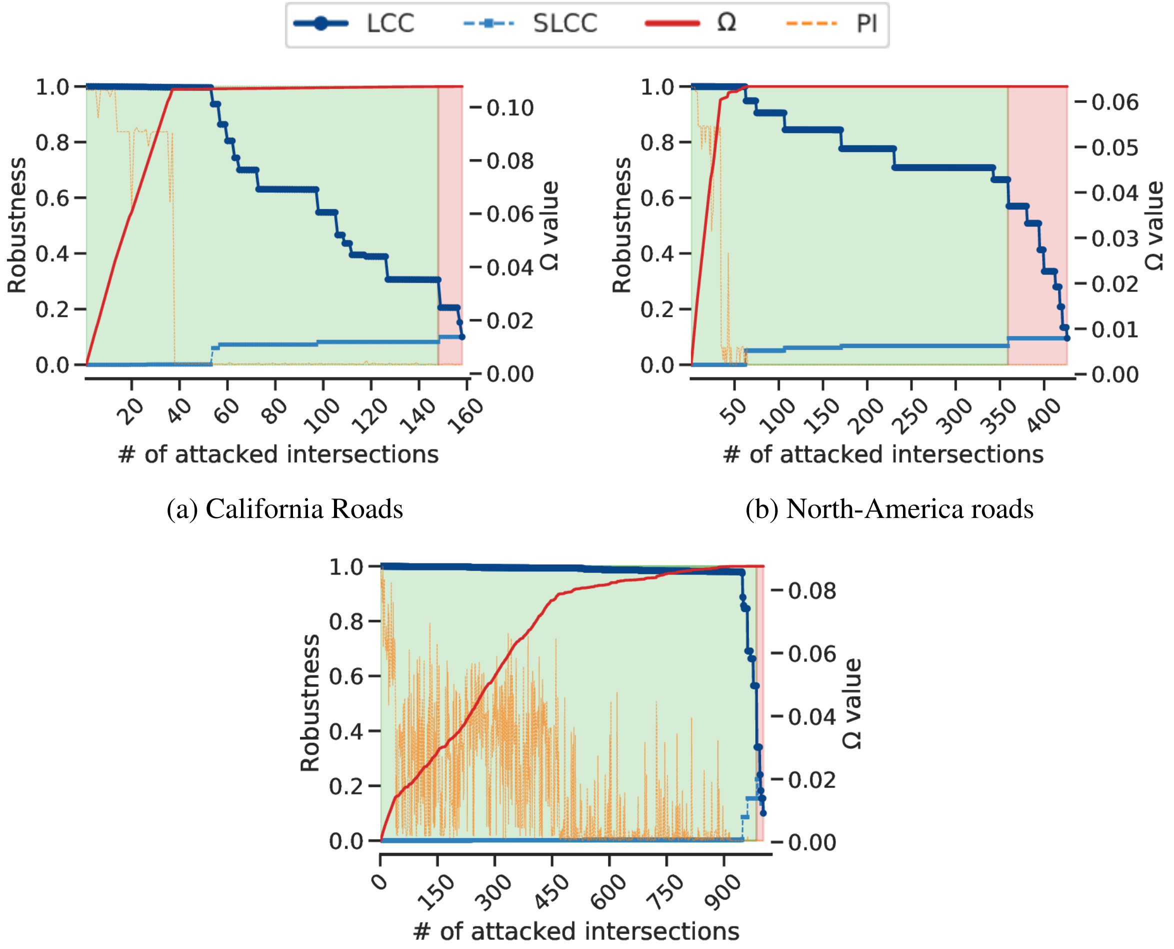

(a) California Roads

(b) North-America roads

(c) San Francisco roads

Supplementary Figure 12: Ω values for three different American road networks under GND +R attacks (with cost matrix $\mathbf{W} = \mathbf{I}$).

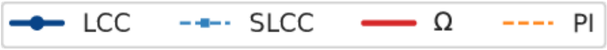

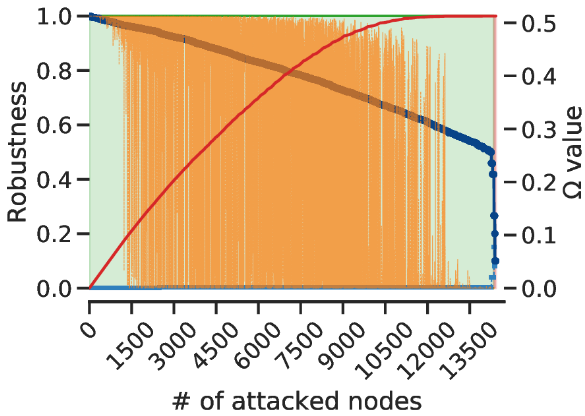

(a) Internet topology (tech-RL-caida)

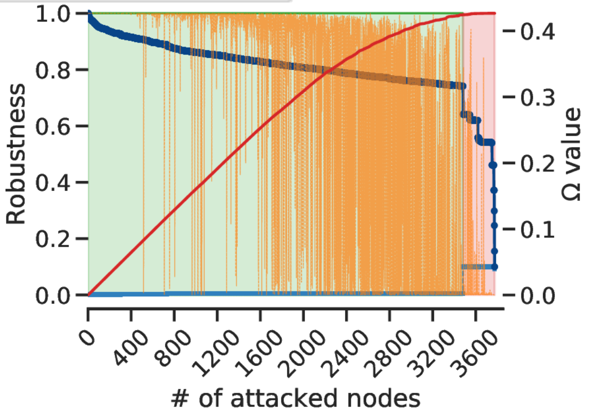

(b) University of Notre Dame website hyperlinks network

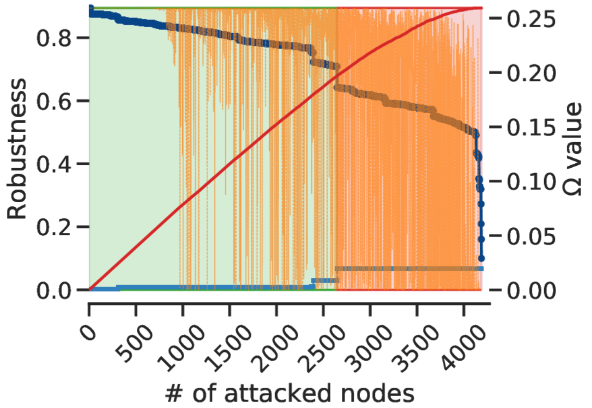

(c) Stanford University website hyperlinks network

Supplementary Figure 13: Ω values for three different internet networks under GND +R attacks (with cost matrix $\mathbf{W} = \mathbf{I}$).

and EI), we use authors' official code with default parameters. In particular, we use identity weight input matrix for both GND and EGND (and the relative fine-tuning algorithm), $1K$ trials for the EGND.

Supplementary References

1. Fey, M. & Lenssen, J. E. Fast graph representation learning with PyTorch Geometric. In *ICLR Workshop on Representation Learning on Graphs and Manifolds* (2019).

2. Paszke, A. *et al.* Automatic differentiation in pytorch (2017).

3. Peixoto, T. P. The graph-tool python library. *figshare* (2014). URL `http://figshare.com/articles/graph_tool/1164194`.

4. Kipf, T. N. & Welling, M. Semi-supervised classification with graph convolutional networks. *arXiv preprint arXiv:1609.02907* (2016).

5. Holme, P., Kim, B. J., Yoon, C. N. & Han, S. K. Attack vulnerability of complex networks. *Phys. Rev. E* **65**, 056109 (2002). URL `https://link.aps.org/doi/10.1103/PhysRevE.65.056109`.

6. Page, L., Brin, S., Motwani, R. & Winograd, T. The pagerank citation ranking: Bringing order to the web. Tech. Rep., Stanford InfoLab (1999).

7. Ying, R., Bourgeois, D., You, J., Zitnik, M. & Leskovec, J. Gnnexplainer: Generating explanations for graph neural networks (2019). `1903.03894`.

8. CAIDA. Ipv4 routed /24 as links dataset. URL `http://www.caida.org/data/active/ipv4_routed_topology_aslinks_dataset.`

9. Advogato network dataset – KONECT (2017). URL `http://konect.cc/networks/advogato`.

10. Massa, P., Salvetti, M. & Tomasoni, D. Bowling alone and trust decline in social network sites. In *Proc. Int. Conf. Dependable, Autonomic and Secure Computing*, 658–663 (2009).

11. Caenorhabditis elegans network dataset – KONECT (2017). URL `http://konect.cc/networks/arenas-meta`.

12. Duch, J. & Arenas, A. Community detection in complex networks using extremal optimization. *Phys. Rev. E* **72**, 027104 (2005).

13. Google.com internal network dataset – KONECT (2017). URL `http://konect.cc/networks/cfinder-google`.

14. Palla, G., Farkas, I. J., Pollner, P., Derényi, I. & Vicsek, T. Directed network modules. *New J. Phys.* **9**, 186 (2007).

15. Citeseer network dataset – KONECT (2017). URL `http://konect.cc/networks/citeseer`.

16. Bollacker, K., Lawrence, S. & Giles, C. L. CiteSeer: An autonomous Web agent for automatic retrieval and identification of interesting publications. In *Proc. Int. Conf. on Autonomous Agents*, 116–123 (1998).

17. Dblp co-authorship network dataset – KONECT (2017). URL `http://konect.cc/networks/com-dblp`.

18. Yang, J. & Leskovec, J. Defining and evaluating network communities based on ground-truth. In *Proc. ACM SIGKDD Workshop on Mining Data Semantics*, 3 (ACM, 2012).

19. Ribeiro, H. V., Alves, L. G. A., Martins, A. F., Lenzi, E. K. & Perc, M. The dynamical structure of political corruption networks. *Journal of Complex Networks* **6**, 989–1003 (2018). URL https://doi.org/10.1093/comnet/cny002. http://oup.prod.sis.lan/comnet/article-pdf/6/6/989/28007544/cny002.p[illegible]

20. Dblp network dataset – KONECT (2017). URL http://konect.cc/networks/dblp-cite.

21. Ley, M. The DBLP computer science bibliography: Evolution, research issues, perspectives. In *Proc. Int. Symposium on String Processing and Information Retrieval*, 1–10 (2002).

22. Digg friends network dataset – KONECT (2017). URL http://konect.cc/networks/digg-friends.

23. Hogg, T. & Lerman, K. Social dynamics of Digg. *EPJ Data Science* **1** (2012).

24. Caenorhabditis elegans (neural) network dataset – KONECT (2018). URL http://konect.cc/networks/dimacs10-celegansneural.

25. Watts, D. J. & Strogatz, S. H. Collective dynamics of 'small-world' networks. *Nature* **393**, 440–442 (1998).

26. White, J. G., Southgate, E., Thomson, J. N. & Brenner, S. The structure of the nervous system of the nematode Caenorhabditis elegans. *Phil. Trans. R. Soc. Lond* **314**, 1–340 (1986).

27. Political blogs network dataset – KONECT (2018). URL http://konect.cc/networks/dimacs10-polblogs.

28. Adamic, L. A. & Glance, N. The political blogosphere and the 2004 US election: Divided they blog. In *Proc. Int. Workshop on Link Discov.*, 36–43 (2005).

29. Douban network dataset – KONECT (2017). URL `http://konect.cc/networks/douban`.

30. Zafarani, R. & Liu, H. Social computing data repository at ASU (2009). URL `http://socialcomputing.asu.edu`.

31. Rossi, R. A. & Ahmed, N. K. The network data repository with interactive graph analytics and visualization. In *AAAI* (2015). URL `http://networkrepository.com`.

32. Twitter lists network dataset – KONECT (2017). URL `http://konect.cc/networks/ego-twitter`.

33. McAuley, J. & Leskovec, J. Learning to discover social circles in ego networks. In *Advances in Neural Information Processing Systems*, 548–556 (2012).

34. Eu institution network dataset – KONECT (2017). URL `http://konect.cc/networks/email-EuAll`.

35. Leskovec, J., Kleinberg, J. & Faloutsos, C. Graph evolution: Densification and shrinking diameters. *ACM Trans. Knowledge Discovery from Data* **1**, 1–40 (2007).

36. Matke, C., Medjroubi, W. & Kleinhans, D. SciGRID - An Open Source Reference Model for the European Transmission Network (v0.2) (2016). URL `http://www.scigrid.de`.

37. Florida ecosystem dry network dataset – KONECT (2017). URL `http://konect.cc/networks/foodweb-baydry`.

38. Ulanowicz, R. E., Heymans, J. J. & Egnotovich, M. S. Network analysis of trophic dynamics in South Florida ecosystems, FY 99: The graminoid ecosystem. *Annual Report to the United States Geological Service Biological Resources Division Ref. No.[UMCES] CBL 00-0176, Chesapeake Biological Laboratory, University of Maryland* (2000).

39. Florida ecosystem wet network dataset – KONECT (2017). URL `http://konect.cc/networks/foodweb-baywet`.

40. Wiegmans, B. Gridkit: European and north-american extracts (2016).

41. Hyves network dataset – KONECT (2017). URL `http://konect.cc/networks/hyves`.

42. Colizza, V., Pastor-Satorras, R. & Vespignani, A. Reaction–diffusion processes and metapopulation models in heterogeneous networks. *Nature Physics* **3**, 276–282 (2007).

43. Batagelj, V. & Mrvar, A. Pajek datasets. URL `http://vlado.fmf.uni-lj.si/pub/networks/data/`.

44. Internet topology network dataset – KONECT (2017). URL `http://konect.cc/networks/topology`.

45. Zhang, B., Liu, R., Massey, D. & Zhang, L. Collecting the Internet AS-level topology. *SIGCOMM Computer Communication Review* **35**, 53–61 (2005).

46. Ciaodvd trust network dataset – KONECT (2018). URL `http://konect.cc/networks/librec-ciaodvd-trust`.

47. Guo, G., Zhang, J., Thalmann, D. & Yorke-Smith, N. ETAF: An extended trust antecedents framework for trust prediction. In *Proc. Int. Conf. Adv. in Soc. Netw. Anal. and Min.*, 540–547 (2014).

48. Filmtrust trust network dataset – KONECT (2018). URL http://konect.cc/networks/librec-filmtrust-trust.

49. Guo, G., Zhang, J. & Yorke-Smith, N. A novel Bayesian similarity measure for recommender systems. In *Proc. Int. Joint Conf. on Artif. Intell.*, 2619–2625 (2013).

50. Linux network dataset – KONECT (2017). URL http://konect.cc/networks/linux.

51. Brightkite network dataset – KONECT (2017). URL http://konect.cc/networks/loc-brightkite_edges.

52. Cho, E., Myers, S. A. & Leskovec, J. Friendship and mobility: User movement in location-based social networks. In *Proc. Int. Conf. on Knowledge Discovery and Data Mining*, 1082–1090 (2011).

53. Gowalla network dataset – KONECT (2017). URL http://konect.cc/networks/loc-gowalla_edges.

54. De Domenico, M., Solé-Ribalta, A., Gómez, S. & Arenas, A. Navigability of interconnected networks under random failures. *Proceedings of the National Academy of Sciences* **111**, 8351–8356 (2014). URL https://www.pnas.org/content/111/23/8351. https://www.pnas.org/content/111/23/8351.full.pdf.

55. Human protein (stelzl) network dataset – KONECT (2017). URL http://konect.cc/networks/maayan-Stelzl.

56. Stelzl, U. *et al.* A human protein–protein interaction network: A resource for annotating the proteome. *Cell* **122**, 957–968 (2005).

57. Human protein (figeys) network dataset – KONECT (2017). URL http://konect.cc/networks/maayan-figeys.

58. Ewing, R. M. *et al.* Large-scale mapping of human protein–protein interactions by mass spectrometry. *Molecular Systems Biology* **3** (2007).

59. Little rock lake network dataset – KONECT (2017). URL http://konect.cc/networks/maayan-foodweb.

60. Martinez, N. D., Magnuson, J. J., Kratz, T. & Sierszen, M. Artifacts or attributes? effects of resolution on the Little Rock Lake food web. *Ecological Monographs* **61**, 367–392 (1991).

61. Human protein (vidal) network dataset – KONECT (2017). URL http://konect.cc/networks/maayan-vidal.

62. Rual, J.-F. *et al.* Towards a proteome-scale map of the human protein–protein interaction network. *Nature* 1173–1178 (2005).

63. Crime network dataset – KONECT (2017). URL http://konect.cc/networks/moreno_crime.

64. Protein network dataset – KONECT (2017). URL http://konect.cc/networks/moreno_propro.

65. Coulomb, S., Bauer, M., Bernard, D. & Marsolier-Kergoat, M.-C. Gene essentiality and the topology of protein interaction networks. *Proceedings of the Royal Society B: Biological Sciences* **272**, 1721–1725 (2005).

66. Han, J.-D. J., Dupuy, D., Bertin, N., Cusick, M. E. & Vidal, M. Effect of sampling on topology predictions of protein-protein interaction networks. *Nature Biotechnology* **23**, 839–844 (2005).

67. Stumpf, M. P., Wiuf, C. & May, R. M. Subnets of scale-free networks are not scale-free: Sampling properties of networks. *Proceedings of the National Academy of Sciences of the United States of America* **102**, 4221–4224 (2005).

68. Train bombing network dataset – KONECT (2017). URL `http://konect.cc/networks/moreno_train`.

69. Hayes, B. Connecting the dots. can the tools of graph theory and social-network studies unravel the next big plot? *American Scientist* **94**, 400–404 (2006).

70. Digg network dataset – KONECT (2017). URL `http://konect.cc/networks/munmun_digg_reply`.

71. Choudhury, M. D., Sundaram, H., John, A. & Seligmann, D. D. Social synchrony: Predicting mimicry of user actions in online social media. In *Proc. Int. Conf. on Comput. Science and Engineering*, 151–158 (2009).

72. Twitter (icwsm) network dataset – KONECT (2017). URL `http://konect.cc/networks/munmun_twitter_social`.

73. Choudhury, M. D. *et al.* How does the data sampling strategy impact the discovery of information diffusion in social media? In *ICWSM*, 34–41 (2010).

74. Openflights network dataset – KONECT (2017). URL `http://konect.cc/networks/opsahl-openflights`.

75. Opsahl, T., Agneessens, F. & Skvoretz, J. Node centrality in weighted networks: Generalizing degree and shortest paths. *Social Networks* **3**, 245–251 (2010).

76. Us power grid network dataset – KONECT (2017). URL `http://konect.cc/networks/opsahl-powergrid`.

77. Uc irvine messages network dataset – KONECT (2017). URL `http://konect.cc/networks/opsahl-ucsocial`.

78. Opsahl, T. & Panzarasa, P. Clustering in weighted networks. *Social Networks* **31**, 155–163 (2009).

79. Leskovec, J., Kleinberg, J. & Faloutsos, C. Graphs over time: Densification laws, shrinking diameters and possible explanations. In *Proceedings of the Eleventh ACM SIGKDD International Conference on Knowledge Discovery in Data Mining*, KDD '05, 177–187 (Association for Computing Machinery, New York, NY, USA, 2005). URL `https://doi.org/10.1145/1081870.1081893`.

80. Ripeanu, M., Foster, I. & Iamnitchi, A. Mapping the Gnutella network: Properties of large-scale peer-to-peer systems and implications for system design. *IEEE Internet Comput. J.* **6** (2002).

81. Leskovec, J. & Krevl, A. SNAP Datasets: Stanford large network dataset collection (2014). URL `http://snap.stanford.edu/data`.

82. Gnutella network dataset – KONECT (2017). URL `http://konect.cc/networks/p2p-Gnutella31`.

83. Erdős network dataset – KONECT (2018). URL `http://konect.cc/networks/pajek-erdos`.

84. Catster/dogster familylinks/friendships network dataset – KONECT (2017). URL http://konect.cc/networks/petster-carnivore.

85. Hamsterster full network dataset – KONECT (2017). URL http://konect.cc/networks/petster-hamster.

86. Li, F., Cheng, D., Hadjieleftheriou, M., Kollios, G. & Teng, S.-H. On trip planning queries in spatial databases. In Bauzer Medeiros, C., Egenhofer, M. J. & Bertino, E. (eds.) *Advances in Spatial and Temporal Databases*, 273–290 (Springer Berlin Heidelberg, Berlin, Heidelberg, 2005).

87. Penn State University Libraries. Digital chart of the world server (2006). URL http://www.maproom.psu.edu/dcw/.

88. Brinkhoff, T. A framework for generating network-based moving objects. *GeoInformatica* **6** (2000).

89. Route views network dataset – KONECT (2017). URL http://konect.cc/networks/as20000102.

90. Slashdot threads network dataset – KONECT (2017). URL http://konect.cc/networks/slashdot-threads.

91. Gómez, V., Kaltenbrunner, A. & López, V. Statistical analysis of the social network and discussion threads in Slashdot. In *Proc. Int. World Wide Web Conf.*, 645–654 (2008).

92. Slashdot zoo network dataset – KONECT (2017). URL http://konect.cc/networks/slashdot-zoo.

93. Kunegis, J., Lommatzsch, A. & Bauckhage, C. The Slashdot Zoo: Mining a social network with negative edges. In *Proc. Int. World Wide Web Conf.*, 741–750 (2009). URL http://cc/ kunegis/paper/kunegis-slashdot-zoo.pdf.

94. Jdk dependency network dataset – KONECT (2016). URL http://konect.cc/networks/subelj_jdk.

95. Jung and javax dependency network dataset – KONECT (2017). URL http://konect.cc/networks/subelj_jung-j.

96. Šubelj, L. & Bajec, M. Software systems through complex networks science: Review, analysis and applications. In *Proc. Int. Workshop on Software Mining*, 9–16 (2012).

97. Morone, F. & Makse, H. A. Influence maximization in complex networks through optimal percolation. *Nature* **524**, 65 (2015).

98. De Nooy, W., Mrvar, A. & Batagelj, V. *Exploratory social network analysis with Pajek*, vol. 27 (Cambridge University Press, 2011).

99. Notre dame network dataset – KONECT (2017). URL http://konect.cc/networks/web-NotreDame.

100. Albert, R., Jeong, H. & Barabási, A.-L. Internet: Diameter of the world-wide web. *Nature* **401**, 130–131 (1999).

101. Stanford network dataset – KONECT (2017). URL http://konect.cc/networks/web-Stanford.

102. Leskovec, J., Lang, K., Dasgupta, A. & Mahoney, M. W. Community structure in large networks: Natural cluster sizes and the absence of large well-defined clusters. *Internet Mathematics* **6**, 29–123 (2009).

103. Boldi, P., Codenotti, B., Santini, M. & Vigna, S. UbiCrawler: A scalable fully distributed web crawler. *Software: Practice & Experience* **34**, 711–726 (2004).

104. Boldi, P., Rosa, M., Santini, M. & Vigna, S. Layered label propagation: A multiresolution coordinate-free ordering for compressing social networks. In *WWW*, 587–596 (2011).

105. Wikipedia links (li) network dataset – KONECT (2018). URL http://konect.cc/networks/wikipedia_link_li.

106. Wikipedia links (kn) network dataset – KONECT (2018). URL http://konect.cc/networks/wikipedia_link_kn.

107. Wordnet network dataset – KONECT (2017). URL http://konect.cc/networks/wordnet-words.

108. Fellbaum, C. (ed.) *WordNet: an Electronic Lexical Database* (MIT Press, 1998).

Network	Name	Category	$\|N\|$	$\|E\|$	References
ARK201012_LCC	CAIDA ARK (Dec 2010) (LCC)	Infrastructure	29.3K	78.1K	(*8*)
advogato	Advogato trust network	Social	6.5K	43.3K	(*9, 10*)
arenas-meta	C. elegans	Metabolic	453	2.0K	(*11, 12*)
cfinder-google	Google.com internal	Hyperlink	15.8K	149.5K	(*13, 14*)
citeseer	CiteSeer	Citation	384.4K	1.7M	(*15, 16*)
com-dblp	DBLP co-authorship	Coauthorship	317.1K	1.0M	(*17, 18*)
corruption	Corruption Scandals	Social	309	3.3K	(*19*)
dblp-cite	DBLP citation	Citation	12.6K	49.6K	(*20, 21*)
digg-friends	Digg friends	Social	279.6K	1.5M	(*22, 23*)
dimacs10-celegansneural	C. elegans (neural)	Neural	297	2.1K	(*24–26*)
dimacs10-polblogs	Political blogs (LCC)	Hyperlink	1.2K	16.7K	(*27, 28*)
douban	Douban social network	Social	154.9K	327.2K	(*29, 30*)
econ-wm1	Economic network WM1	Economic	260	2.6K	(*31*)
ego-twitter	Twitter lists	Social	23.4K	32.8K	(*32, 33*)
email-EuAll	EU institution email	Communication	265.2K	365.6K	(*34, 35*)
eu-powergrid	SciGRID Power Europe	Power	1.5K	1.8K	(*36*)
foodweb-baydry	Florida ecosystem dry	Trophic	128	2.1K	(*37, 38*)
foodweb-baywet	Florida ecosystem wet	Trophic	128	2.1K	(*38, 39*)
gridkit-eupowergrid	GridKit Power Europe	Power	13.8K	17.3K	(*40*)
gridkit-north_america	GridKit Power North-America	Power	16.2K	20.2K	(*40*)
hyves	Hyves social network	Social	1.4M	2.8M	(*30, 41*)
inf-USAir97	US Air lines (1997)	Infrastructure	332	2.1K	(*31, 42, 43*)
internet-topology	Internet (AS) topology	Infrastructure	34.8K	107.7K	(*44, 45*)
librec-ciaodvd-trust	CiaoDVD trust network	Social	4.7K	33.1K	(*46, 47*)
librec-filmtrust-trust	FilmTrust trust network	Social	874	1.3K	(*48, 49*)
linux	Linux source code files	Software	30.8K	213.7K	(*50*)
loc-brightkite	Brightkite friendships	Social	58.2K	214.1K	(*51, 52*)
loc-gowalla	Gowalla friendships	Social	196.6K	950.3K	(*52, 53*)
london_transport_multiplex_aggr	Aggregated London Transportation network	Transport	369	430	(*54*)
maayan-Stelzl	Human protein (Stelzl)	Metabolic	1.7K	3.2K	(*55, 56*)
maayan-figeys	Human protein (Figeys)	Metabolic	2.2K	6.4K	(*57, 58*)
maayan-foodweb	Little Rock Lake food web	Trophic	183	2.5K	(*59, 60*)
maayan-vidal	Human protein (Vidal)	Metabolic	3.1K	6.7K	(*61, 62*)
moreno_crime_projected	Crime (projection)	Social	754	2.1K	(*63*)
moreno_propro	Protein	Metabolic	1.9K	2.3K	(*64–67*)
moreno_train	Train bombing terrorist contacts	Human contact	64	243	(*68, 69*)
munmun_digg_reply_LCC	Digg social network replies (LCC)	Communication	29.7K	84.8K	(*70, 71*)
munmun_twitter_social	Twitter follows (ICWSM)	Social	465.0K	833.5K	(*72, 73*)
opsahl-openflights	OpenFlights	Infrastructure	2.9K	15.7K	(*74, 75*)
opsahl-powergrid	US power grid	Infrastructure	4.9K	6.6K	(*25, 76*)
opsahl-ucsocial	UC Irvine messages	Communication	1.9K	13.8K	(*77, 78*)
oregon2_010526	Autonomous systems Oregon-2	Infrastructure	11.5K	32.7K	(*79*)
p2p-Gnutella06	Gnutella P2P, August 8 2002	Computer	8.7K	31.5K	(*80, 81*)
p2p-Gnutella31	Gnutella P2P, August 31 2002	Computer	62.6K	147.9K	(*80, 82*)
pajek-erdos	Erdős co-authorship network	Coauthorship	6.9K	11.8K	(*43, 83*)
petster-catdog-household	Catster/Dogster familylinks (LCC)	Social	324.9K	2.6M	(*84*)
petster-hamster	Hamsterster full	Social	2.4K	16.6K	(*85*)
power-eris1176	Power network problem	Power	1.2K	9.9K	(*31*)
roads-california	California Road Network	Infrastructure	21.0K	21.7K	(*86*)
roads-northamerica	North-America Road Network	Infrastructure	175.8K	179.1K	(*87*)
roads-sanfrancisco	San Francisco Road Network	Infrastructure	175.0K	221.8K	(*88*)
route-views	Autonomous systems AS-733	Infrastructure	6.5K	13.9K	(*35, 89*)
slashdot-threads	Slashdot threads	Communication	51.1K	117.4K	(*90, 91*)
slashdot-zoo	Slashdot Zoo	Social	79.1K	467.7K	(*92, 93*)
subelj_jdk	JDK dependency network	Software	6.4K	53.7K	(*94*)
subelj_jung-j	JUNG and Javax dependency network	Software	6.1K	50.3K	(*95, 96*)
tech-RL-caida	Internet router network	Infrastructure	190.9K	607.6K	(*31*)
twitter_LCC	Twitter users (LCC)	Social	532.3K	694.6K	(*97*)
web-EPA	Pages linking to epa.gov	Hyperlink	4.3K	8.9K	(*31, 98*)
web-NotreDame	Notre Dame web pages	Hyperlink	325.7K	1.1M	(*99, 100*)
web-Stanford	Stanford University web pages	Hyperlink	281.9K	2M	(*101, 102*)
web-webbase-2001	Web network	Hyperlink	16.1K	25.6K	(*31, 103, 104*)
wikipedia_link_kn	Wikipedia links (KN)	Hyperlink	29.5K	278.7K	(*105*)
wikipedia_link_li	Wikipedia links (LI)	Hyperlink	49.1K	294.3K	(*106*)
wordnet-words	WordNet lexical network	Lexical	146.0K	657.0K	(*107, 108*)

Supplementary Table 5: The networks used to evaluate our approach. For each network, we report the name, the number of nodes and edges, the category it belongs to and some references.
